# Lactic Acidosis Triggers Starvation Response with Paradoxical Induction of TXNIP through MondoA

**DOI:** 10.1371/journal.pgen.1001093

**Published:** 2010-09-02

**Authors:** Julia Ling-Yu Chen, Daniel Merl, Christopher W. Peterson, Jianli Wu, Patrick Yantyng Liu, Hanwei Yin, Deborah M. Muoio, Don E. Ayer, Mike West, Jen-Tsan Chi

**Affiliations:** 1 Institute for Genome Sciences and Policy, Duke University, Durham, North Carolina, United States of America; 2 Department of Molecular Genetics and Microbiology, Duke University, Durham, North Carolina, United States of America; 3 Department of Statistical Science, Duke University, Durham, North Carolina, United States of America; 4 Huntsman Cancer Institute, Department of Oncological Sciences, University of Utah, Salt Lake City, Utah, United States of America; 5 Department of Medicine, National Yang-Ming University, Taipei, Taiwan; 6 Sarah W. Stedman Nutrition and Metabolism Center, Department of Pharmacology and Cancer Biology and Department of Medicine, Duke University Medical Center, Durham, North Carolina, United States of America; Georgia Institute of Technology, United States of America

## Abstract

Although lactic acidosis is a prominent feature of solid tumors, we still have limited understanding of the mechanisms by which lactic acidosis influences metabolic phenotypes of cancer cells. We compared global transcriptional responses of breast cancer cells in response to three distinct tumor microenvironmental stresses: lactic acidosis, glucose deprivation, and hypoxia. We found that lactic acidosis and glucose deprivation trigger highly similar transcriptional responses, each inducing features of starvation response. In contrast to their comparable effects on gene expression, lactic acidosis and glucose deprivation have opposing effects on glucose uptake. This divergence of metabolic responses in the context of highly similar transcriptional responses allows the identification of a small subset of genes that are regulated in opposite directions by these two conditions. Among these selected genes, TXNIP and its paralogue ARRDC4 are both induced under lactic acidosis and repressed with glucose deprivation. This induction of TXNIP under lactic acidosis is caused by the activation of the glucose-sensing helix-loop-helix transcriptional complex MondoA:Mlx, which is usually triggered upon glucose exposure. Therefore, the upregulation of TXNIP significantly contributes to inhibition of tumor glycolytic phenotypes under lactic acidosis. Expression levels of TXNIP and ARRDC4 in human cancers are also highly correlated with predicted lactic acidosis pathway activities and associated with favorable clinical outcomes. Lactic acidosis triggers features of starvation response while activating the glucose-sensing MondoA-TXNIP pathways and contributing to the “anti-Warburg” metabolic effects and anti-tumor properties of cancer cells. These results stem from integrative analysis of transcriptome and metabolic response data under various tumor microenvironmental stresses and open new paths to explore how these stresses influence phenotypic and metabolic adaptations in human cancers.

## Author Summary

Solid tumors usually have many differences in their chemical environments, such as low oxygen, depletion of glucose, high acidity (low pH), and accumulation of lactate, from normal tissues. These changes are usually called tumor microenvironmental stresses. In this study, we have used microarrays to compare the transcriptional response and metabolic adaptation in response to these different stresses seen in the tumor microenvironments. Through these comparisons, we have found that lactic acidosis triggers a starvation response, highly similar to glucose deprivation, even in the presence of abundant nutrients and oxygen. Even the cells seem to be starved; cells under lactic acidosis have decreased glucose uptake. We found this unexpected biological behavior was due to the paradoxical induction of a glucose-sensing Mondo-TXNIP pathway. The activation of this novel anti-tumor pathway under lactic acidosis contributes to the anti-Warburg effect and the restriction of cell growth in tumorigenesis by limiting nutrient availability and its inactivation may be required for tumor progression under these microenvironmental stresses.

## Introduction

Human cancers are extremely heterogeneous due to multiple mutations in oncogenes and tumor suppressor genes, a range of inherited germline variations and varying degrees of microenvironmental stresses. These tumor microenvironmental stresses include tumor hypoxia, accumulation of lactic acid (lactic acidosis) and depletion of glucose, glutamine and other nutrients [1]. These stresses are largely caused by a combination of poor tissue perfusion, abnormal tumor vasculature, uncontrolled proliferation and dysregulated energy metabolism of cancer cells during tumor development and progression. Importantly, these microenvironmental stresses also directly modulate physiological and metabolic phenotypes of cancer cells and ultimately affect the clinical outcomes of patients. With major variations known to exist among different tumors, advances in the pretreatment assessment of the influences of these stresses will aid in improved selection of appropriate therapeutic strategies for individual patients. These stresses and their downstream effects are also the targets of cancer therapeutics, including anti-angiogenesis and hyperthermia treatments. It is therefore important to fully understand the impact and mechanism of how these stresses affect various tumor and non-tumor cells in human cancers.

It is well known that cells resort to glycolysis instead of oxidative phosphorylation to utilize glucose as energy source during hypoxia. In addition, cancer cells have a preferential use of glycolysis pathways for energy generation even in the presence of oxygen – so called “aerobic glycolysis” as first proposed by Dr. Otto Warburg [2]. These factors all likely contribute to high glucose flux and form the basis of using glucose analog ^18^F-FDG to detect tumor cells. Such dysregulated metabolisms in cancer cells also lead to the accumulation of the metabolic product of glycolysis – lactic acids in solid tumors. Many measurements have been performed to determine the level of tumor lactate and significant variations were found, with the medium range of 7–10 mM/g and up to 25.9 mM/g [3]–[5]. These studies show that high tumor lactate levels are typically associated with more aggressive tumors and resistance to treatment [3]–[5]. How lactic acidosis affects tumor and non-tumor cells in human cancers has been the focus of many elegant studies as summarized in several reviews [6]–[8]. The exposure of cultured cells to lactic acidosis *in vitro* has been shown to trigger calcium signaling [9], induce angiogensis gene expression (e.g. VEGF, IL8) [10]–[12], HIF-1α stabilization [13] and cell death [14]. Recent genomic analyses identify the transcriptional responses of different cell types to acidosis and high lactate [15]–[17]. These *in vitro* studies have clearly shown the significant impact of lactic acidosis on the gene expression and phenotypes of cancer cells.

However, it is often challenging to relate the influences of these microenvironmental stresses *in vitro* to the complex cancer phenotypes *in vivo*. We have previously overcome this challenge by defining “gene signatures” based on sets of genes whose expression levels are altered by lactic acidosis *in vitro* as quantitative “common phenotypes”, and projecting them to the *in vivo* microarray expression data of patients' tumors [18]. We have used this approach to successfully investigate the pathways of hypoxia [19], vascular injury [20] and lactic acidosis [18] in human cancers. This reciprocal flow of information between the *in vitro* and *in vivo* systems demonstrate that lactic acidosis triggers significant metabolic reprogramming by forcing cells to rely more on oxidative phosphorylation as an energy source with the suppression of glycolytic phenotypes [18]. Therefore, cellular metabolism is critical in determining the impact of lactic acidosis to tumor phenotypes and clinical outcomes.

In the use of microarrays to connect cultured cells with human cancers, primary epithelial cells are often used to provide the common and shared responses to the defined perturbations due to their intact genetic materials and signaling circuitry [18]–[22]. However, given our intention to relate gene signatures to human cancers, it is probably more relevant to assess signatures in cancer cells. Cancer cell lines are also often easier to be transfected, thus allowing genetic manipulations for mechanistic studies. In this study, we used breast cancer cell line, MCF-7, to conduct a detailed temporal analysis of lactic acidosis response and compare the transcriptomic and metabolic responses of cancer cells to hypoxia, lactic acidosis and glucose deprivation in parallel to gain a further understanding of the underlying molecular mechanisms.

One important goal in understanding the cellular responses to tumor microenvironmental stresses is to identify the key regulator(s) responsible for these observed gene expression response since such understanding will lead to important insights into the development and progression of human cancers. For example, the identification of hypoxia-inducible transcription factors (HIFs) as central regulators in the hypoxia response and its regulation at the level of protein stability has become crucial in our understanding of tumor hypoxia [23]–[26]. HIF-1α protein stabilization can also be seen in the development of multiple neoplasms in patients with von Hippel-Lindau disease [27] or mutations in several enzymes of the tricarboxylic acid (TCA) cycle, such as succinate dehydrogenase (SDH) and fumarate hydratase (FH) [28]–[30]. The glucose deprivation is another feature of tumor microenvironmental stress caused by the imbalance between supply and consumption [31]. Glucose deprivation triggers “starvation-like” signaling through the activation of AMPK and LKB-1, which in turn activates TSC1/TSC2 and inhibits central energy sensor mTOR activities to inhibit ribosomal biogenesis, translation activities and proliferation [32]. The mutations in LKB-1 and TSCs in the glucose sensing pathway lead to cancer development in the Peutz-Jeghers Cancer Syndrome [33] and Tuberous Sclerosis [34], [35], respectively. In contrast, very little is known about the transcriptional regulation of the lactic acidosis gene response program. To uncover the molecular mechanisms by which tumor cells respond to their microenvironment, we conducted a detailed temporal transcriptional analysis of the MCF7 breast cancer cell line under lactic acidosis and compared this response to those elicited by glucose deprivation and hypoxia. We identified a novel growth suppressive pathway (MondoA-TXNIP) that portends better prognosis in breast cancer and contribute to the anti-Warburg effects of lactic acidosis.

## Results

### The temporal transcriptional responses of MCF-7 under lactic acidosis

We examined the temporal gene expression patterns of a breast cancer cell line MCF-7 exposed to lactic acidosis (25 mM lactic acid with pH 6.7) at various time points during the first 24 hours. Cells were first brought to replicative arrest by serum withdrawal for 24 hours before exposure to either control or lactic acidosis conditions composed of pH 6.7 and 25mM lactic acid in the presence of high levels of glucose (4.5 g/L) in triplicate of different time points at 1, 4, 12 and 24 hrs to characterize the temporal changes of gene expression patterns. The RNA samples harvested from these MCF-7 cells were interrogated with Affymetrix GeneChip U133 plus 2.0 arrays (∼54,000 probe sets on ∼47,000 transcripts and variants) with results deposited in Gene Expression Omnibus (GSE19123).

**Figure 1:**
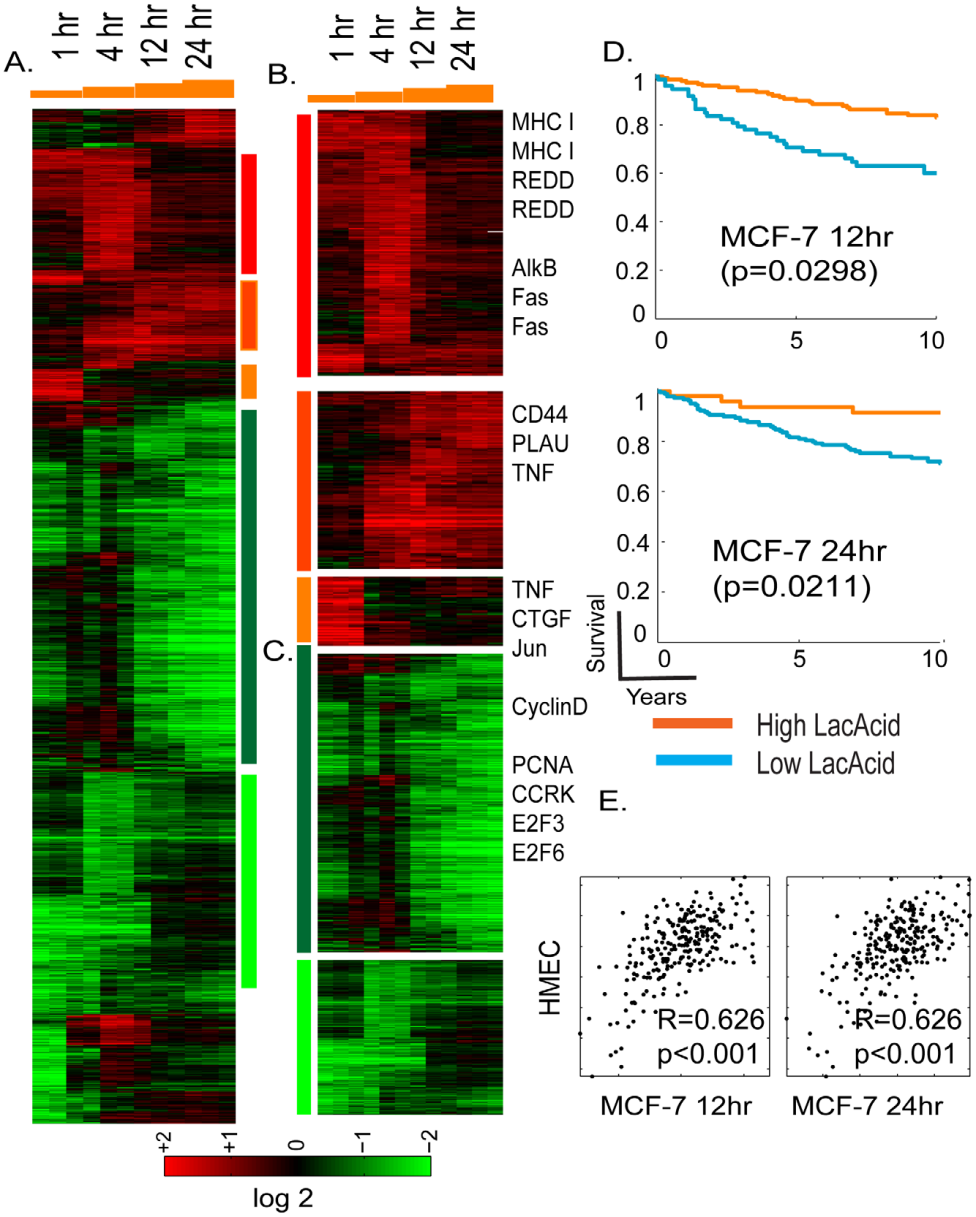
Overview of the time course of lactic acidosis response in MCF-7. (A) The gene expression response of MCF-7 is shown when exposed to lactic acidosis conditions at indicated time points. 1761 probes sets were selected by the criteria of at least two observations with at least two fold changes and arranged by hierarchical clustering. Clusters of genes induced by different time points and repressed by lactic acidosis are marked and further expanded in (B), and (C) with the names of selected genes shown. (D) The prognostic significance of the lactic acidosis pathway activity in MCF-7 at 12 and 24 hours were assessed in the Miller breast cancer expression dataset. The tumors, stratified by the imputed signature scores associated with the LA response, were used to generate Kaplan-Meier survival curves linking clinical outcomes with the indicated responses. (E) Scatter plots showing the relationship between the estimated lactic acidosis pathway activities using the pathway signature obtained in HMEC (Y-axis) vs. the signature obtained in MCF-7 (X-axis) at 12 and 24 hours. Each point in the scatter plot represents a single tumor from the indicated breast cancer data set. The overall correlation (R) and statistical significance/p-value (p) between the lactic acidosis signature scores elicited in MCF-7 and HMECs across all samples is shown the data set.

Gene expression profiles of all MCF-7 cells were normalized by RMA, zero-transformed against the average expression levels of the same probe sets of the time-matched control samples as performed previously [19], [20]. 1761 probes sets showing with at least two fold changes in at least two samples were selected and arranged by hierarchical clustering according to similarities in expression patterns (Figure 1A). This analysis showed that lactic acidosis induced a dramatic change in the gene expression with significant temporal patterns (Figure 1A and 1B).

Among the genes induced in MCF-7 by lactic acidosis, we found PLAU, major histocompatility complex (MHC) type I and CD44, and REDD1 (Figure 1B), a p53 transcriptional target following DNA damage [36]. The expression of CD44 has been reported to be induced by lactosis in cancer cells [37]. Tumor necrosis factor (TNF), Fas and connective tissue growth factors (CTGF) were only induced in earlier time points and returned to baseline in the later time points (Figure 1B). Among clusters of genes repressed by lactic acidosis in the later time points is a large group whose expression is closely linked to cell proliferation (cyclin D, PCNA, CCRK, E2F3, and E2F6) (Figure 1C). These lactic acidosis-repressed genes may reflect a physiological alteration that halts cell proliferation as the cells try to preserve energy consumption under metabolic stress [31], [32].

To define the lactic acidosis response in MCF-7 at a pathway level, we performed Gene Set Enrichment Analysis (GSEA) [38] to compare the pathway composition in the gene expression of all control vs. lactic acidosis samples of MCF-7 cells. We found that samples exposed to lactic acidosis were enriched in gene sets representing nutrient deprivation [39], the treatment of histone deacetylase inhibitor trichostatin A (TSA) in cancer cells, breast cancer good prognosis [40] and exposure of DNA damaging agent (bleomycin) (Table S1). The gene expression of MCF-7 which has been exposed to lactic acidosis was depleted in gene sets representing E2F1 target genes, DNA replication, breast cancer poor prognosis [40], mitotic cycles and RNA processing (Table S1).

We previously showed that the hypoxia and lactic acidosis response signatures elicited in cultured primary non-cancerous cells HMECs could provide a molecular gauge of hypoxia and lactic acidosis response for cancerous human tissues *in vivo*, and also predict clinical outcomes. To test robustness of this gene signature approach based now on cancerous cells, the lactic acidosis gene signatures generated in MCF-7 were projected to the Miller breast cancer data to evaluate a tumor-specific numerical score representing the predicted signature of lactic acidosis – i.e., quantifying lactic acidosis pathway activity across tumors [41]; see Materials and Methods section below and the detailed statistical supplemental materials from a previous study [18]. Statistical survival analysis then indicates that patients with tumors showing higher levels of lactic acidosis pathway activity – defined by projected signatures of 12 and 24 hours exposure in MCF-7 – had significantly better clinical outcomes (Figure 1D), consistent with our previous studies using HMEC [18]. The lactic acidosis gene signature at 12 hour time point had the most consistent prognostic significance across different tumor datasets, including three other breast cancer expression studies with different stages of diseases (Figure S1). These datasets include a study of 286 lymph node negative early breast cancers from NKI (Wang) [42], and two studies of invasive breast carcinomas (Sotiriou, Pawitan) [43], [44].

We also measured the reproducibility and consistency of the predicted lactic acidosis pathway activities in each tumor using gene signatures generated in both MCF-7 and HMECs. We found that the two variants of the lactic acidosis signature resulted in highly similar predicted lactic acidosis activity, evidenced by strongly correlated signature scores in Miller and other tumor datasets (Figure 1E and Figure S2). This result suggests that although there are differences between the lactic acidosis responses at the gene level, the pathway level predictions based on the gene signatures are similar and reproducible in both normal primary and breast cancer cells.

### Lactic acidosis triggers “starvation” response

**Figure 2:**
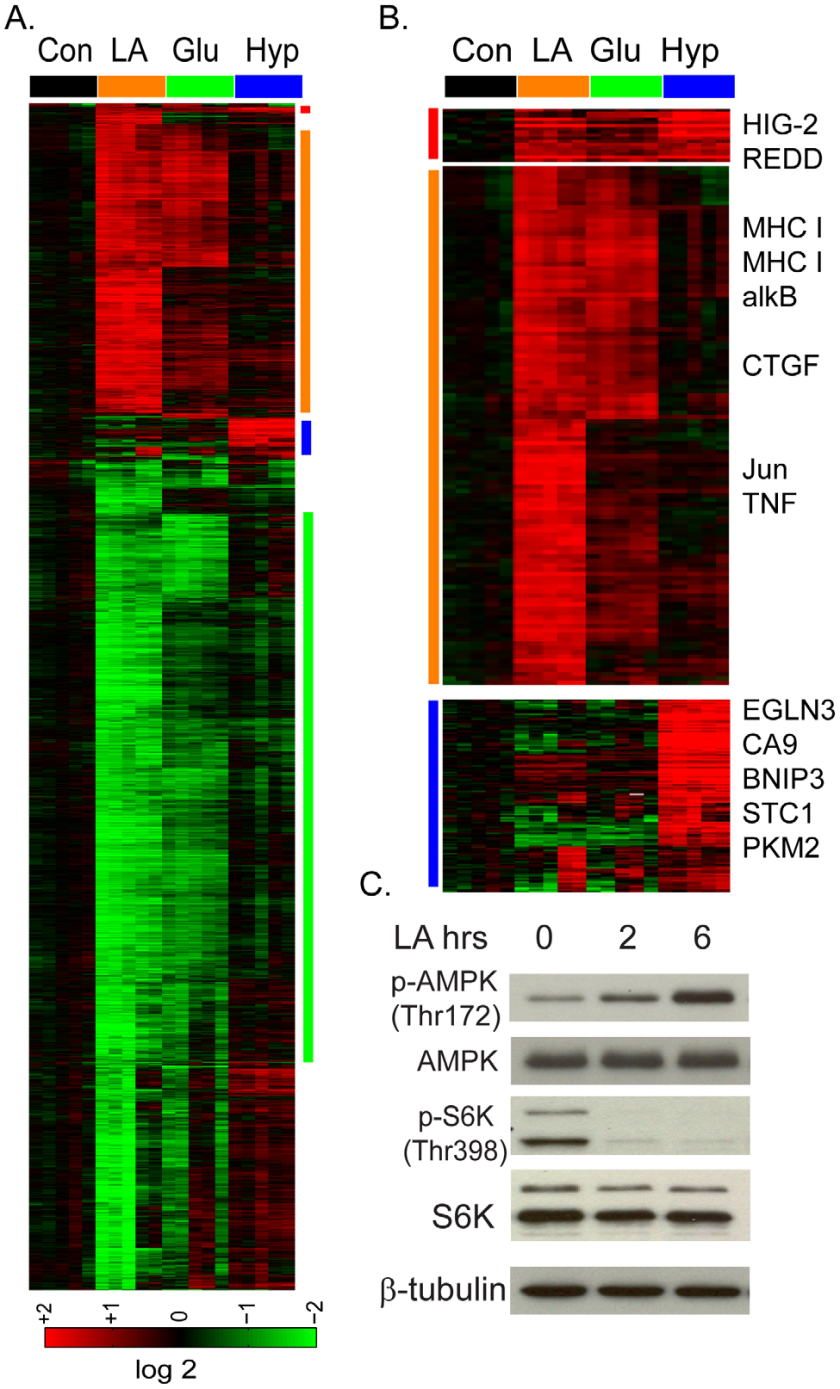
The lactic acidosis triggers starvation response. (A) The transcriptional response of MCF-7 to the lactic acidosis, glucose deprivation and hypoxia at four hours. Selected gene clusters which were induced commonly by lactic acidosis and glucose deprivation and hypoxia, by lactic acidosis and glucose deprivation, or by hypoxia alone were highlighted and expanded in (B) with selected names shown. (C) Lactic acidosis triggers the activation of AMPK (phosphorylation at Thr 172) and the inhibition of mTORC1 as manifested by the reduction of S6K phosphorylation at Thr 398.

In the GSEA analysis for the MCF-7 cells exposed to lactic acidosis, we found enrichment in the starvation pathways caused by the deprivation of glucose and glutamine obtained in an independent study [39]. Such association between lactic acidosis and nutrient deprivation was consistent with the reduced ATP production under lactic acidosis [18]. To test for such association, we directly compared the gene expression of MCF-7 cells of lactic acidosis with cellular starvation stress caused by glucose deprivation. We performed parallel global transcriptional analysis on the MCF7 cells in quintuplicate which had been exposed to lactic acidosis and 1% oxygen (hypoxia) in the media with high glucose (4.5g/L) and glucose deprivation (using media 0g/L glucose) for 4 hours. Similar parallel analysis was performed for subsets of genes involved in metastasis and invasive capacity [45], [46]. RNAs from these MCF-7 cells were extracted and hybridized to Affymetrix GeneChip U133 plus 2.0 arrays, normalized by RMA and zero-transformed by deducting the mean expression values for samples under control condition. 3903 probe sets were selected by the criteria of at least two observations with at least two fold changes and arranged by hierarchically clustering (Figure 2A and 2B). Unexpectedly, we noted a highly similar transcriptional response to lactic acidosis and glucose deprivation which is distinct from the hypoxia response (Figure 2A). The similarity of the lactic acidosis and glucose deprivation response was observed in both induction and repression of a large number of common sets of genes, including major histocompatility complex (MHC) type I & II, DNA repair gene, alkB (alkyation repair homolog 7), and CTGF, Jun and TNF (Figure 2B), which were also seen in our previous time course experiment (Figure 1B). In contrast, hypoxia elicited a very distinct transcriptional response with the upregulation of many known hypoxia-inducible genes such as EGLN3, CA9, stanniocalcin1 (STC1), BNIP3 and genes involved in glycolysis, including pyruvate kinase (PKM2) (Figure 2B) [19]. In a previous microarray study of the stress response in yeast *Saccharomyces cerevisiae*, the induction of a share set of “common stress genes” was a prominent feature [47]. In contrast, there was only a small cluster of genes which were induced by lactic acidosis, glucose deprivation and hypoxia, which may represent “common stress genes” in MCF-7 cells (Figure 2B). These genes include HIG-2 (hypoxia-inducible gene) and REDD, both genes reported to be induced by hypoxia (Figure 2B).

Since glucose deprivation is known to trigger cellular starvation response, this similarity in the transcriptional responses suggests that lactic acidosis may also trigger a “starvation” response seen for nutrient deprivation. To test this possibility, we measured the effects of lactic acidosis on several biochemical markers of starvation response in MCF-7 (Figure 2C). AMP-activated protein kinase (AMPK) is a highly conserved energy-sensing heterotrimeric complex that plays a key role in the regulation of energy homeostasis; it becomes phosphorylated at Thr172 of AMPK α by an elevated AMP/ATP ratio due to various stress indicating energy stress and starvation. We find that lactic acidosis significantly increased Thr172 phosphorylation of AMPK, even in the presence of high levels of glucose and amino acids in the media (Figure 2C). The Mammalian Target of Rapamcyin (mTOR) is another crucial cellular sensor for energy status and a crucial downstream target of AMPK [48], [49]. We examined how lactic acidosis affected mTORC1 activities through the phosphorylation of its downstream target S6 kinase (S6K) and found it dramatically reduced the S6K phophorylation (Figure 2C). Taken together, these data supported the notion that lactic acidosis triggers a cellular starvation response similar to that of glucose starvation, with AMPK activation and mTOR inhibition. These biochemical changes are likely to contribute to reduced cell growth and proliferation under lactic acidosis as evidenced by gene expression (Figure 1C).

### The distinct metabolic effects of lactic acidosis and glucose deprivation

**Figure 3:**
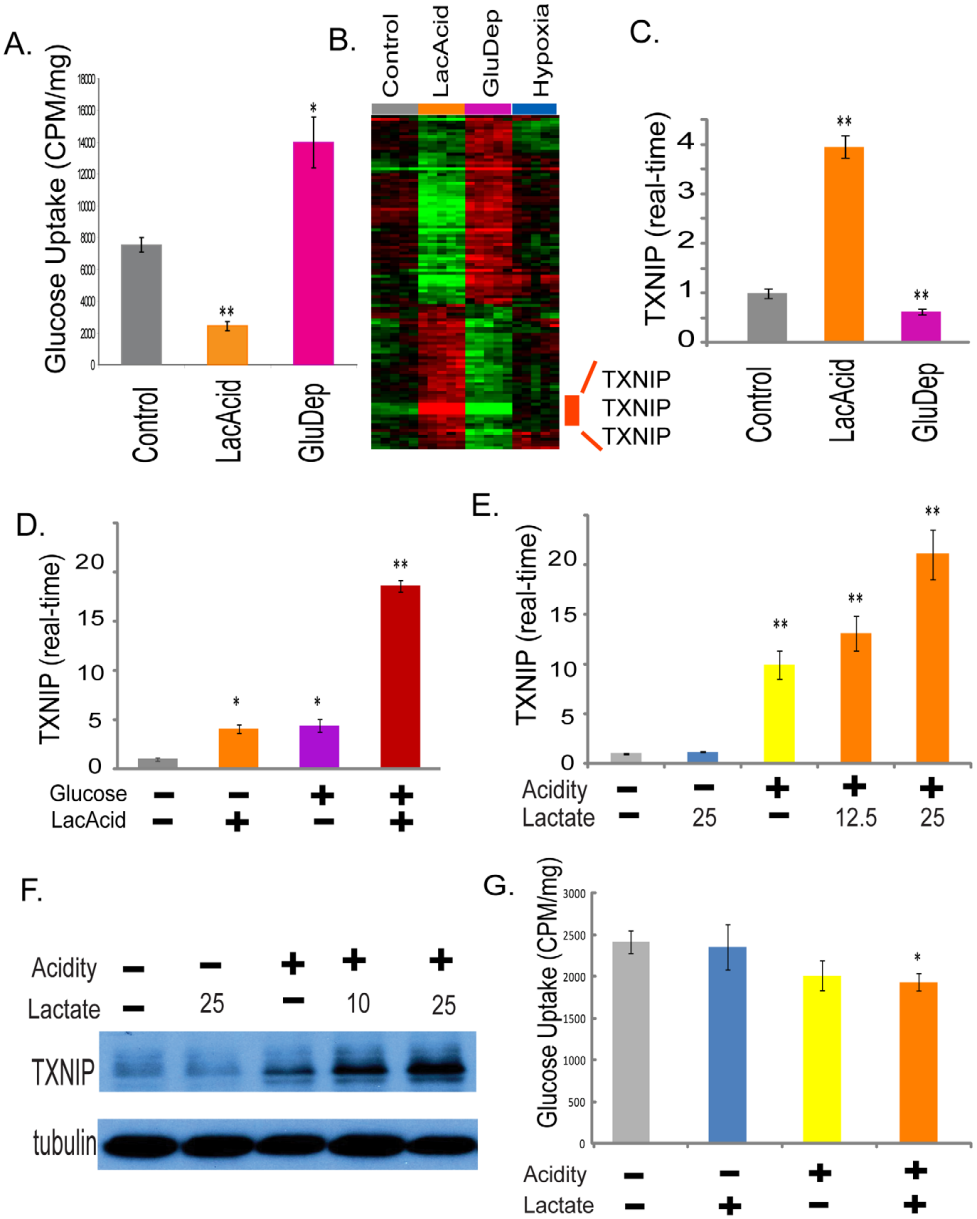
The induction of TXNIP under lactic acidosis. (A) The amount of glucose uptake of the MCF-7 under control, lactic acidosis and glucose deprivation conditions. (B) Heat map shows expression of the 115 selected probe sets in MCF-7 placed in control, lactic acidosis, glucose deprivation and hypoxia for four hours with the probe sets for TXNIP highlighted. (C) The level of TXNIP transcripts determined by real-time PCR in the MCF-7 under indicated conditions. (D) The level of TXNIP transcripts determined by real-time PCR in the MCF-7 under the four indicated conditions for lactic acidosis and glucose level. (E, F) The level of TXNIP determined by real-time PCR (E) and Western blot (F) in the MCF-7 under five indicated conditions with or without acidity (pH 6.7) and carrying levels of lactate (10, 12.5 or 25 mM). (G) The amount of glucose uptake of the MCF-7 under control, acidosis, lactosis (25mM) and lactic acidosis (25mM) conditions.

Knowing that lactic acidosis can inhibit tumor glycolysis [18], we evaluated how lactic acidosis and glucose deprivation affect glucose uptake using 2-deoxy-D-[2,6-^3^H]-glucose. While glucose deprivation increased the uptake of the glucose by 84%, lactic acidosis significantly reduced the glucose uptake by 67% (Figure 3A). The inhibition of glucose uptake by lactic acidosis was consistent with the reduced glucose consumption and decreased lactate production we have reported previously. However, this result was not consistent with the expected increased uptake of glucose to increase energy generation associated with AMPK activation and cellular starvation, as seen in glucose deprivation [50]. Therefore, in spite of the shared AMPK activation, mTORC1 inhibition and similar transcriptional responses, lactic acidosis and glucose starvation triggered opposite effects on glucose uptake.

To identify transcriptionally regulated genes that may contribute to these opposite metabolic responses, we used the discordance to select genes with significant but opposite expression changes under lactic acidosis and glucose deprivation. This analysis identified 115 probe sets (Table S2); 49 were induced by lactic acidosis and repressed by glucose deprivation while 66 were repressed by lactic acidosis and induced by glucose deprivation (Figure 3B). The three probe sets with the largest induction levels under lactic acidosis were all associated with thioredoxin interacting protein (TXNIP or Vitamin D3-upregulated protein 1, VDUP1) (Figure 3B). TXNIP was strongly induced by lactic acidosis, and suppressed by glucose deprivation. While hypoxia was reported to trigger TXNIP expression [51], we only noted a modest increase (∼40%) in our experiment. We tested for the prognostic significance of this differentially expressed signature by projecting the 115 gene signature into the Miller breast tumor data set. Due to different arrays used in that data set, this results in 109 of the 115 signature probes present in the Miller data set. We find that higher levels of this signature also indicate favorable outcomes (Figure S3A). The three TXNIP probe sets alone in fact have prognostic significance comparable with that of the signature (Figure S3B); on re-evaluating the signature after removal of these three TXNIP probe sets, we find that the reduced signature maintains prognostic significance albeit at a slightly reduced level (Figure S3C).

When we examined expression of MCF-7 and HMEC under lactic acidosis at different time points, we also noted significant induction of TXNIP and its parralogue alpha-arrestin domain containing 4 (ARRDC4) (Figure S4). Real-time RT-PCR further confirmed the induction of TXNIP (up to 4–5 fold) and ARRDC4 transcripts (up to 3.4 fold) in MCF-7 (Figure 3C and Figure S5). In addition, lactic acidosis also induced TXNIP expression in other cancer cell lines, including WiDr (colon cancer cell) and SiHa (cervical cancer cell) (Figure S6).

TXNIP expression is known to be induced by glucose exposure [52], [53]. Since there were high levels of glucose (4.5g/dL) in both control and lactic acidosis media, we further clarified the role of glucose vs. lactic acidosis in the TXNIP induction using real-time PCR to determine the level of TXNIP in MCF-7 cultured under different glucose and lactic acidosis conditions (Figure 3D). Glucose (4.5g/dL) or lactic acidosis alone increased TXNIP levels by approximately 4–5 fold (Figure 3D). In the presence of both high glucose levels and lactic acidosis, TXNIP was induced up to 18 fold (Figure 3D). Thus lactic acidosis and glucose exposure are both potent inducers of TXNIP with synergistic induction potential, suggesting distinct mechanisms of TXNIP induction by these two stimuli.

To define the individual contribution of lactosis vs. acidosis, we tested the level of TXNIP induction under different degrees of lactosis (12.5 or 25 mM lactate) and acidosis (pH 6.7) (Figure 3E and 3F). We found that acidosis alone, but not lactosis alone, led to the TXNIP induction, consistent with our previous study [18] (Figure 3E). Although lactosis alone did not induce TXNIP, the addition of lactate to the acidosis conditions led to a dose-dependent augmentation of the TXNIP induction (Figure 3E). Importantly, this effect on TXNIP induction was present even under 10 or 12.5 mM lactate, a level seen in many human tumors (Figure 3E and 3F). We also measured how lactosis and acidosis affected glucose uptake, and found that acidosis, but not lactosis, led to a reduction in glucose uptake, a pattern consistent with TXNIP induction (Figure 3G).

**Figure 4:**
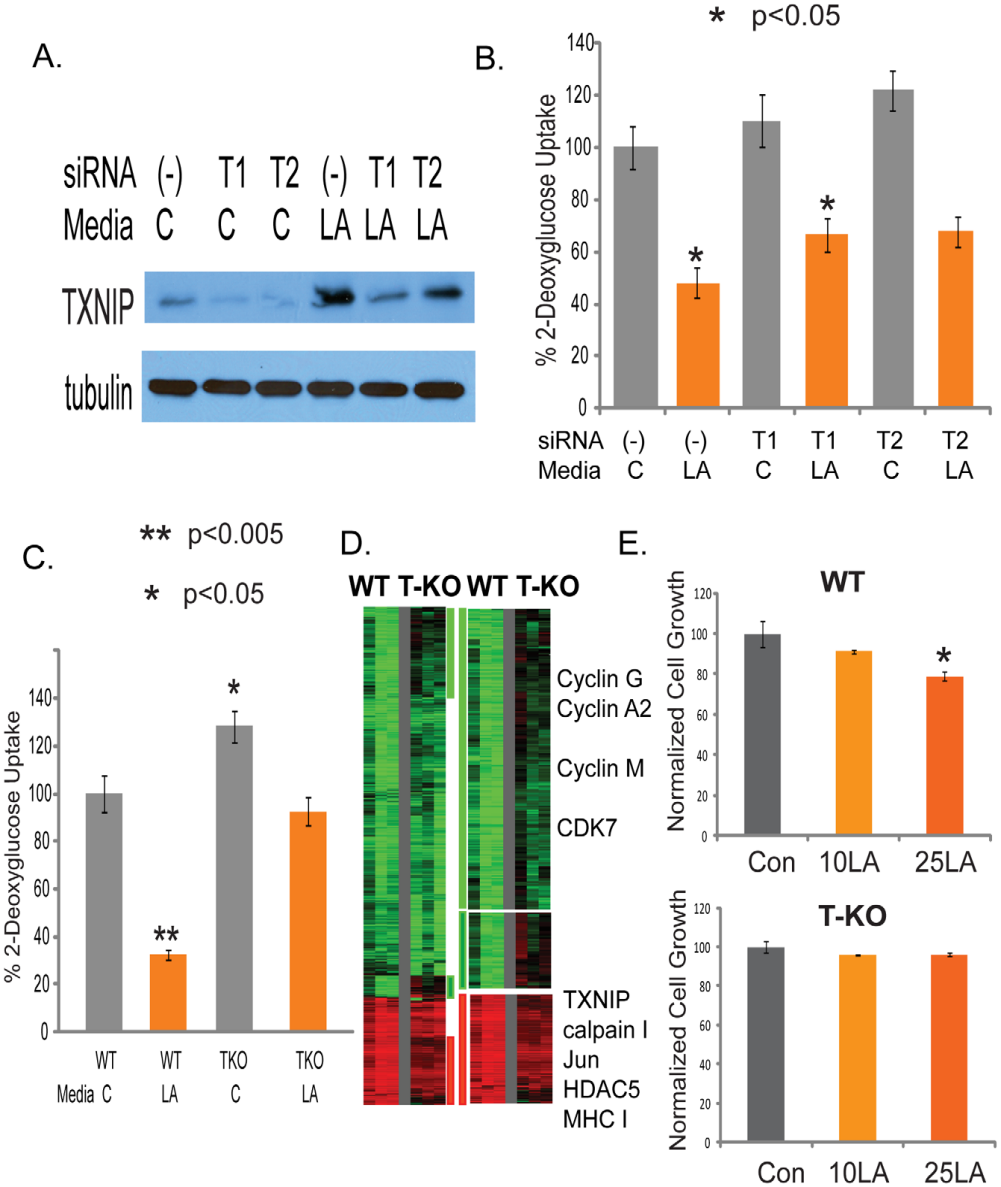
Identification of TXNIP as a regulator of lactic acidosis response. (A) The level of TXNIP proteins treated with control or siRNAs against TXNIP under control or lactic acidosis conditions. (B) The level of glucose uptake in the MCF-7 treated with the indicated conditions. Lactic acidosis caused 52% repression of glucose uptake in MCF7 cells transfected with non-targeting siRNAs (-) as negative control. In cells transfected with two different siTXNIPs (T1, T2), glucose uptake was increased and the repressing effect of lactic acidosis was decreased to 39% and 44% respectively. (C) Lactic acidosis caused 68% repression in wild-type (WT) MEF cells but only 28% repression in TXNIP knockout (TKO) MEF cells. (D) The gene expression response of wild type and TXNIP deficient MEF cells was shown when exposed to 10mM lactic acidosis conditions. 1327 probes sets showing with at least 1.7 fold changes in at least two samples were selected and arranged by hierarchical clustering according to similarities in expression patterns. Clusters of genes whose induction and repression was most affected by TXNIP are marked and further expanded with the names of selected genes shown. (E) The effect of the control and lactic acidosis (10mM LA and 25mM LA) on the cell growth in percentage of the wild type and TXNIP deficient MEF cells.

Although TXNIP was initially identified as a protein interacting with thioredoxin and modulating cellular responses to oxidative stresses, it has the ability to inhibit glucose uptake and is being recognized as an important regulator of dysregulated metabolism in diabetes [54], [55]. Therefore, the upregulation of TXNIP under lactic acidosis make it an attractive candidate contributing to the anti-Warburg effects and inhibition of tumor glycolysis under lactic acidosis [18]. To investigate this, we silenced the TXNIP transcripts with two independent siRNAs and confirmed the successful reduction of the TXNIP protein (Figure 4A). Consistent with the previously known role of TXNIP to inhibit glucose uptake [55], the silencing of TXNIP by siRNAs led to significant decrease in the reduction of glucose uptake under lactic acidosis culture condition (Figure 4B). Lactic acidosis caused 52% repression of glucose uptake in MCF7 cells transfected with non-targeting siRNAs (-). In cells transfected with two different siTXNIPs (T1, T2), glucose uptake was increased while the repressing effect of lactic acidosis was decreased to 39% and 44% respectively (Figure 4B). In addition, we found the silencing of TXNIP in MCF-7 increased both the glucose consumption and lactate productions under normal media, but lactic acidosis significantly reduced both the glucose consumption and lactate production in all treated cells (Figure S7).

Although the TXNIP gene silencing reduced the glycolysis inhibition under lactic acidosis, the effects were modest, which may be due to the remaining level of TXNIP. We further tested the effect of TXNIP knocking out in the TXNIP deficient MEF cells [56]. Lactic acidosis caused 68% reduction in glucose uptake in the wild-type (WT) MEF cells. In contrasts, lactic acidosis only reduced the glucose uptake by 28% in TXNIP knockout (TKO) MEF cells (Figure 4C). To further test for the role of TXNIP upregulation in the lactic acidosis response, we exposed the wild type and TXNIP deficient MEF cells to the lactic acidosis conditions (10 mM lactate, pH 6.7) and performed gene expression analysis using the Affymetrix mouse 430A2 GeneChip and normalized the expression data by RMA. We first examined the effects of the disruption of TXNIP on the gene expression of MEF cells by performing zero transformation of TXNIP deficient against the average expression in the wild type MEF cells (Figure S8). We found 798 probe sets whose expression was altered at least 1.7 fold in at least two samples (Figure S8). Among the genes repressed in the TXNIP KO MEF cells were TXNIP, COX2, collagens and many other genes in the HOX genes involved in pattern specification (Figure S8). Among the induced in the TXNIP KO MEF cells were genes in the complement activation, AP2, and Notch3 (Figure S8).

To define the role of TXNIP in the lactic acidosis gene expression, we compared the respective lactic acidosis response of the wild type and TXNIP deficient MEF cells by zero-transformation against the corresponding cells cultured in the control conditions. 1327 probe sets showing with at least 1.7 fold changes in at least two samples were selected and arranged by hierarchical clustering according to similarities in expression patterns (Figure 4D). This analysis showed that the loss of TXNIP reduced the induction and repression of a significant number of genes as shown in the three gene clusters (Figure 4D), including the repression of many cell-cycle related genes under lactic acidosis. In addition, the loss of TXNIP led to a statistically significant reduction in the overall degrees of induction and repression (p<0.0001) for the average expression of the 1049 repressed and 278 induced genes (Figure S9A and Figure 9B). We also tested the ability of lactic acidosis to repress the proliferation of MEF cells and found that the proliferation of the wild type, but not TXNIP deficient MEF cells, were repressed by lactic acidosis (Figure 4E). This observation is consistent with the reduced repression of cell cycle genes under lactic acidosis seen for the TXNIP deficient MEF cells (Figure 4D). Taken together, these data showed that TXNIP induction contributes to the inhibition of glycolysis phenotypes, metabolic reprogramming, and cell cycle arrest and gene expression under lactic acidosis.

### The role of the MondoA:Mlx complex in the activation of TXNIP by lactic acidosis

**Figure 5:**
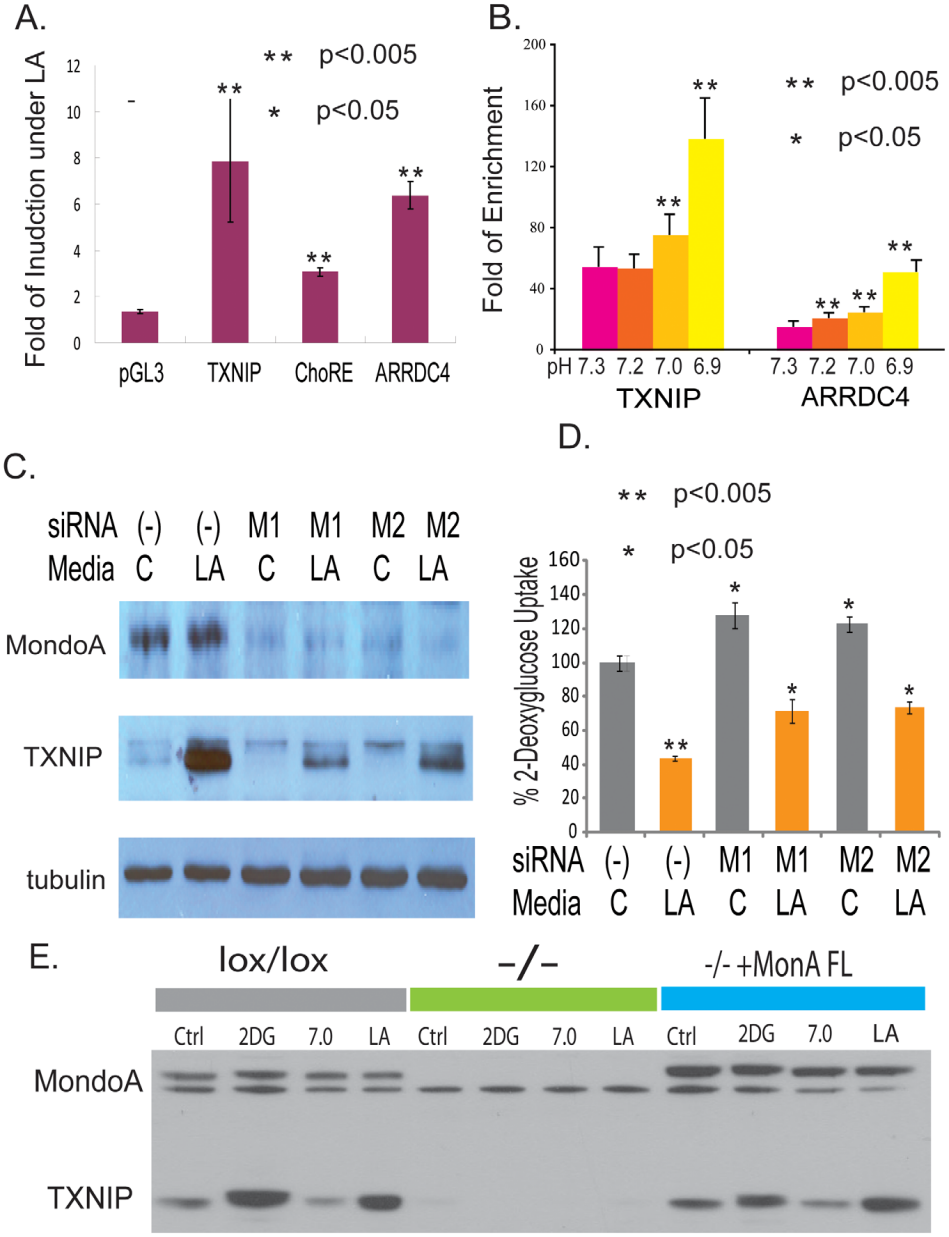
MondoA is responsible for the TXNIP induction under lactic acidosis. (A) The fold of induction of normalized luciferase activities under lactic acidosis for the indicated reporter constructs driven by wild type TXNIP promoter, TXNIP promoter with the ChoRE mutated and ARRDC4 promoters. (B) The physical binding of MondoA to the promoters of TXNIP and ARRDC4 was assessed by Chromatin-Immunoprecipitation for MCF-7 cells under lactic acidosis with different indicated pH. (C) The level of MondoA and TXNIP proteins in MCF-7 cells treated with control or two siRNAs against MondoA under control or lactic acidosis conditions. (D) The level of glucose uptake in the MCF-7 treated with the indicated conditions. Lactic acidosis caused 57% repression in MCF7 cells transfected with non-targeting siRNA (-). The repression effect of lactic acidosis was decreased to 44% and 40% with MCF7 cells transfected with two different MondoA siRNAs (M1 and M2). (E) The level of MondoA and TXNIP proteins shown by Western in the indicated mouse embryonic fibroblasts (MEF): lox/lox (MEF with wild type MondoA), −/− (lox/lox MEF with cre overexpression to delete MondoA) and −/−+MondoA FL = −/− reconstituted with FL human MondoA under control, 2-DG, pH 7 and lactic acidosis conditions.

Glucose-induced TXNIP transcription depends on a short proximal region of the TXNIP promoter. Specifically, this region includes a well conserved Carbohydrate Response Elements (ChoRE) consisting of two E-boxes [53]. We first tested the influence of lactic acidosis on the reporter constructs driven by the promoters of TXNIP and ARRDC4 and found that lactic acidosis could induce the reporter activities of both constructs by more than 6 folds (Figure 5A). Importantly this induction of reporter activity under lactic acidosis was reduced by 61% with mutation in the ChoRE of the TXNIP promoter (Figure 5A), indicating the importance of the ChoRE regions to the transcriptional activation of TXNIP under lactic acidosis.

The transcriptional activation through two E-boxes in the promoters of TXNIP upon glucose exposure has been reported to be caused by the binding MondoA:Mlx [57] or Carbohydrate Response Elements-binding protein (ChREBP) [58]. MondoA is likely to be more relevant in the observed lactic acidosis response of MCF-7 given its higher expression levels in MCF-7 and the simultaneous upregulation of both TXNIP and ARRDC4 under lactic acidosis [57]. MondoA:Mlx complexes are held latently at the outer mitochondrial membrane (OMM), yet shuttle between the OMM and the nucleus suggesting that they facilitate communication between these two essential organelles. MondoA:Mlx complexes are sensors of intracellular glucose concentration and accumulate in the nucleus following increases in glucose-6 phosphate to occupy the E-box-containing promoters of targets such as TXNIP and ARRDC4 [57]. To directly test the inducible physical binding of MondoA to the promoter regions of TXNIP and ARRDC4 under lactic acidosis, chromatin-immunoprecipitation (CHIP) was performed with the antibody against MondoA in MCF-7 cells which have been exposed to lactic acidosis conditions of different acidity. We detected increased specific binding of MondoA to the promoter regions of TXNIP and ARRDC4 with more acidic lactic acidosis environments (Figure 5B). These results indicated that the MonoA became activated to occupy the promoters of TXNIP and ARRDC4 under lactic acidosis and these binding sites were important for their transcriptional inductions.

To further determine the role of MondoA in the induction of TXNIP under lactic acidosis, we used two different sets of siRNAs to knock down MondoA by gene silencing (Figure 5C). During lactic acidosis, the level of MondoA protein did not change, while TXNIP was induced significantly (Figure 5C). This induction of TXNIP was significantly reduced at both levels when MondoA was reduced by both sets of siRNAs targeting MondoA (Figure 5C). The silencing of MondoA also led to increased glucose uptake under both control and lactic acidosis condition (Figure 5D), a result similar to the silencing of TXNIP (Figure 3F and 3H). While lactic acidosis caused 57% repression in MCF7 cells transfected with non-targeting siRNA, such repression was reduced to 44% and 40% with MCF7 cells transfected with two different siRNAs targeting MondoA (M1 and M2). The degree of the lactic acidosis-induced repression of the glucose uptake was lower after the silencing of MondoA by two independent siRNAs (Figure S10).

Although gene silencing led to significant reduction of MondoA, the remaining MondoA level may still account for the slight induction of TXNIP under lactic acidosis. To further examine the effects in the absence of MondoA, we tested the MondoA knockout MEF (mouse embryonic fibroblasts) cells created by the cre-loxP system (CWP and DEA, manuscript submitted). In the complete absence of MondoA, both the basal and inducible level of TXNIP under 2-DG or lactic acidosis was completed abolished (Figure 5E). Importantly, the re-introduction of full length MondoA back to the MEF cells fully restored the induction of TXNIP under lactic acidosis (Figure 5E). Taken together, these results demonstrate the critical role of MondoA in regulating TXNIP under both glucose exposure and lactic acidosis.

### TXNIP expression and lactic acidosis pathways in human cancers

**Figure 6:**
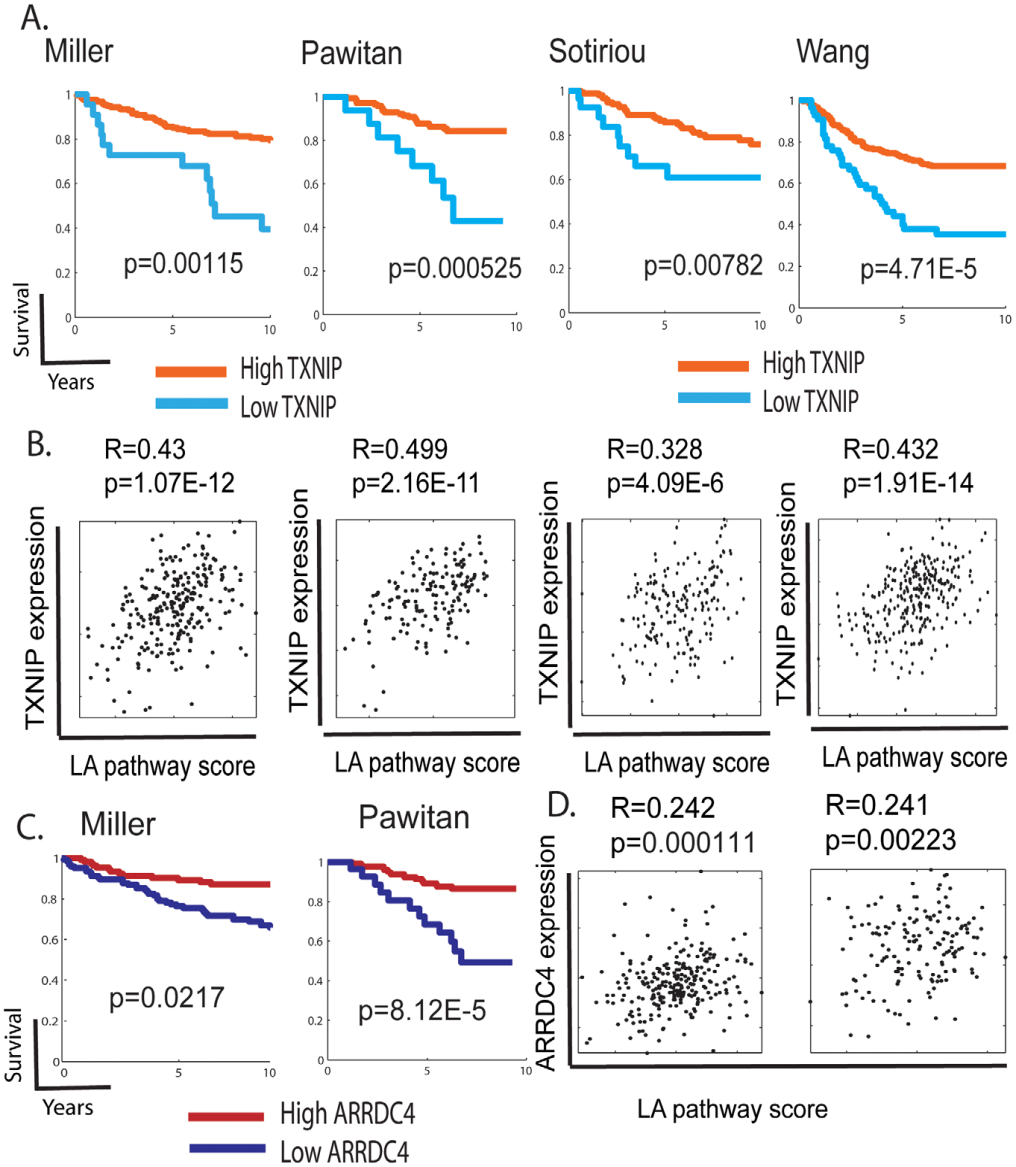
The expression of TXNIP, ARRDC4, and lactic acidosis pathways in human cancers. (A,C) The tumors in the indicated dataset stratified by the expression of TXNIP (A) and ARRDC4 (B) were used to generate Kaplan-Meier survival curves for linking clinical outcomes with the TXNIP expression levels. (B,D) Scatter plots showing the relationship between the expression of TXNIP (B) or ARRDC4 (D) (Y-axis) and predicted lactic acidosis pathway activities based on the MCF-7 12 hour lactic acidosis gene signatures (X-axis) in the indicated tumor datasets. Each point in the scatter plots represents a single tumor from the indicated breast cancer data sets. The overall correlation (R) and statistical significance/p-value (p) across all samples is shown.

To determine the prognostic significance of TXNIP expression in human cancers, we performed survival analyses using TXNIP expression as the sole predictor of survival time. Breast cancers in the Miller dataset stratified based on TXNIP expression were found to have significant differences in clinical phenotypes; tumors with high TXNIP have better survival and clinical outcomes (Figure 6A, p = 0.00115). Similar results were also obtained in the three other breast cancer datasets. To test whether the *in vitro* correlation of TXNIP induction with lactic acidosis pathway activity persists *in vivo*, we compared the predicted lactic acidosis pathway activity with the TXNIP expression levels in the breast cancer data sets and found a positive correlation in all four data sets; although the relationship is of limited predictive value with low R values reflecting a noisy relationship, the positive relationship is statistically significant and consistent across tumor data sets (Figure 6B). Similar results were found for ARRDC4, another paralouge of TXNIP and downstream target of MondoA. When breast cancers were stratified by the level of ARRDC4, tumors with higher levels of ARRDC4 had better prognosis and clinical outcome in the both breast cancer data (Miller and Pawitan) in which ARRDC4 was measured (Figure 6C). The expression of ARRDC4 was also positively associated with the predicted lactic acidosis pathway activity in these two breast cancer datasets (Figure 6D). These observations demonstrate the high degree of *in vivo* correlation between the expression of TXNIP and ARRDC4 and the lactic acidosis pathway activity in human cancers. It is also consistent with the known role of TXNIP as a probable tumor suppressor in several cancer types where it has been shown to suppress oncogenic phenotypes and its loss of function linked with cancer development [59]–[61]. Therefore, the expression of both TXNIP and ARRDC4 are potentially important mediators of the lactic acidosis response and suggest the important prognostic significance of molecular pathways driven by Mondo-Mlx in human cancers.

## Discussion

Hypoxia, glucose deprivation and lactic acidosis are all well-recognized tumor microenvironmental stresses. Each of these stresses has distinct features – either limited availability of energy fuel (glucose), depletion of cofactor (oxygen) or accumulation of metabolic byproducts (lactic acidosis). Previous studies in various settings have shown that glucose deprivation causes metabolic stress and induces adaptive responses —so-called “starvation response” – manifest by AMPK activation, mTOR inhibition and multiple other biochemical and metabolic changes in cancer cells. Tumor hypoxia has also been reported to induce such metabolic stresses [62]. Here, we have shown that lactic acidosis triggers a “starvation” response similar to glucose deprivation as evidenced by the biochemical and transcriptional responses. Since this occurs even in the presence of abundant oxygen and nutrients, this response may represent “pseudo-starvation”. This is especially unexpected given the presence of high levels of lactate as an additional potential energy source for cancer cells [63].

Adaptive changes during the starvation response often reflect a need to preserve energy homeostasis during energy depletion by switching off cell growth and proliferation and many anabolic processes, such as protein, carbohydrate and lipid biosynthesis. In addition, there is also a simultaneous increase in cellular catabolism and breakdown of various energy sources to increase energy production. Even with this shared energy need, different tumor microenvironmental stresses employ different means to accomplish such adaptations to preserve energy homesostasis. During hypoxia, the essential cofactor (oxygen) required for oxidative phosphorylation becomes limited. As a result, the transcriptional factor HIF-1α activates genes encoding glucose transporters and enzymes involved in the glycolytic process shift to the use of glycolysis as the main source of energy generation. In the setting of glucose deprivation, there is a lack of fuel required for glycolysis as energy source. During these energy shortages, AMPK become activated to modify many proteins controlling energy flow to increase the energy generation in mitochondria from other source of fuels, such as oxidative of fatty acid and amino acids [64]. In addition, the inhibition of mTOR turns off many energy-consumption processes (e.g., translation, cell growth and proliferations) in an effort to restore energy homeostasis. The lactic acidosis-induced cell cycle arrest may be caused by many changes, including the induction of AMPK, mTOR inhibition, TXNIP and cell cycle arrest caused by the induction p57, p21 and other inhibitors of cellular proliferation. Moreover, the expression of many genes involved in glycolysis is affected by changes in histone acetylation of chromatin [65]. These responses triggered by glucose deprivation may be also important for energy maintenance and survival in acidosis-induced apoptosis [66], [67]. For example, AMPK may help in re-directing cells to utilize non-glucose energy sources (e.g., fatty acids and amino acids) and increased mitochondria activities under lactic acidosis [18], [68]. In addition, since the AMPK activator AICAR reduces the expression of lactate importer monocarboxylate transporter (MCT)-1 but increases the expression of the lactate exporter in MCT4 [69], AMPK activation under lactic acidosis may also inhibit the uptake of excessive cellular lactate. These stress environments are also likely to select for tumor cells that have developed strategies to survive energy deprivation with better energetic balance. The genetic mutations of many genes involved in these processes of adaptation to hypoxia and glucose deprivation are known to associate with tumor development. These findings highlight the crucial role of biochemical, metabolic control of energy homeostasis in tumor development.

There are many possible explanations as to how lactic acidosis leads to AMPK activation, mTOR inhibition and other features of the starvation response. For example, lactic acidosis can reduce ATP generation in cells [18] and thus lead to a high AMP/ATP ratio to activate the AMPK. Extracellular acidosis also triggers an increase in cytosolic Ca^++^ [9] which may activate CaMKK – an alternative AMPK upstream kinase [70]–[72] – to phophorylate and activate AMPK. Lactic acidosis may also cause intracellular nutrient depletion with reduced uptake of glucose (this study) and glutamine through the inhibition of acidosis-sensitive glutamine pumps [73], [74]. This depletion of intracellular pools of nutrients may in turn repress the energy sensor mTOR, the translation activities and ribosomal biogenesis required for cellular proliferation. In the future, it will be important to further dissect the contribution of each factor and signaling components to the induction of these starvation responses under lactic acidosis to further our detailed understanding of their impact on the metabolisms and phenotypes of cancer cells.

In both hypoxia and glucose deprivation, there is an increase in glucose uptake and glycolysis to provide essential fuel through induction (by HIFs), modification of glucose transporters (by AMPK) [75], [76] and histone acetylation via ATP-citrate lyase [65]. In contrast, lactic acidosis presents a different instance of starvation with inhibition of glucose uptake and other glycolysis activities in cancer cells [77]. Through these comparisons, we have also dissected two distinct molecular pathways (AMPK-mTORC1, MondoA-TXNIP) by which various microenvironmental stresses influence cancer metabolic phenotypes. While the AMPK-mTOR response is similar under lactic acidosis and glucose deprivation, the MondoA-TXNIP is affected in opposite directions by these two stresses. Given the continuous need for energy generation with reduced ATP and metabolic substrate from the glycolysis pathways under lactic acidosis, cells are likely to undergo extensive metabolic reprogramming to utilize other nutrients as energy sources. This idea is supported by the increased reliance on mitochondria for ATP generation. Our analysis has highlighted the potential roles of TXNIP and AMPK in this metabolic reprogramming. The induction of TXNIP under lactic acidosis and its ability to inhibit glucose uptake and reduce lactic acidosis production from glycolysis form a negative feedback loop. In addition, the loss of TXNIP leads to many features of Warburg effects and glycolytic phenotypes of cancer cells, opposite to the influences of lactic acidosis [18]. High TXNIP expression is associated with favorable outcomes, consistent with its postulated role as a tumor suppressor gene based on its growth-suppressing activity and the increased occurrences of tumors with deficiency of TXNIP [59], [60], [78], [79]. These findings may provide important insights into the regulatory mechanisms of TXNIP as well as the phenotypic alterations under lactic acidosis. TXNIP may affect the glucose uptake through the suppression of two important regulators of glycolysis, Akt [56] and HIF-1α [80]. In addition, TXNIP is known for other properties, which may explain its effect on the gene expression during lactic acidosis. TXNIP is a negative regulator of cellular oxidative tolerance by binding thioredoxin [81]–[83] and as a feedback regulator of S-nitrosylation [84] relevant in the cellular adaptive response to tumor microenvironmental stresses. For example, repression of TXNIP enhancing the anti-oxidative capacity during glucose deprivation may be required to cope with increased mitochondria oxidative stresses [85]. 2-Deoxy-D-glucose (2-DG) can enhance the cytotoxicity of cisplatin through mechanisms involving increased oxidative stress [86]. Since 2-DG is a strong inducer of TXNIP [57], a lower anti-oxidative capacity caused by 2-DG exposure may also involve neutralization of the anti-oxidative capacity of thioredoxin by TXNIP induction.

TXNIP and ARRDC4 are both transcriptionally regulated by the MondoA:Mlx complex to coordinate the fuel status of cellular metabolism and proliferation [57], [87]. Our findings identify lactic acidosis as a novel stimulus for the activation of MondoA and induction of TXNIP/ARRDC4. Even though we have identified the importance of MondoA-TXNIP of one component of the lactic acidosis response, there are still many aspects of lactic acidosis which remained unexplained, such as similarities to the glucose deprivation response. At least two factors are likely to be relevant for the shared gene expression response of glucose deprivation and lactic acidosis. First, these changes may lead to significant changes in the level of acetyl-CoA, which in turn impacts on histone acetylation of the chromatin of many target genes [65]. In addition, AMPK activation under both stresses may also impact on several transcription factors and co-activators to affect gene expression [88], [89]. It is likely that the anti-tumor activities of AMPK activators (e.g., metformin or AICAR) [90]–[93] may involve similar pathways triggered by lactic acidosis.

Many studies on the lactate levels in human cancers have found that tumors with high lactate levels are associated with poorer clinical outcomes, tumor aggression and treatment failure [3]–[5]. How do our finding of the association of the lactic acidosis response with favorable outcomes reconcile with these studies? There are at least four different ways how our results can be reconciled with the clinical observation. First, our studies mainly focus on the cellular response of cancer cells to short term exposure of lactic acidosis. This result may be different from the long term selection in the high lactic acidosis environments of human tumors. For example, glucose deprivation triggers starvation response with AMPK activation, mTOR inhibition and cell cycle arrest during short term exposure [48]. During the long term exposure to these stresses in human cancers, these undesirable conditions may select for tumor cells with the somatic mutations (such as K-Ras mutation [94]) that confers ability to adapt these stress conditions and strong metastasis potential. Similar selection pressure has also been suggested for the lactic acidosis [1], [15], [95]. In addition, the high level of lactate in the tumors is the downstream effects of the preferential use of glycolysis pathways due to tumor hypoxia, which may lead to more aggressive tumor behaviors and worse clinical outcomes. While lactic acidosis itself may exert some anti-tumor influences, this may not be enough to counter the influence of these somatic mutations and hypoxia in driving the tumor aggressiveness. It is also important to point out that the experimental evidence suggests that the lactic acidosis response is mainly due to acidosis instead of lactosis. Although we have found that lactosis may further augment the acidosis response, lactosis by itself have relatively little effects on the gene expression. Although the degree of the cellular response to these stresses (such as CA9) can reflect the levels of stresses (low tumor pO_2_) and serve as “endogenous markers” of such stresses in human cancers, such connection are not absolute and direct [96]. Therefore, the degree of lactic acidosis response in human cancers may or may not correlate directly with the tumor lactate levels.

Through the dissection of individual stresses in vitro, we have shown here that lactic acidosis simultaneously triggers two anti-tumor pathways (AMPK-mTOR and MondoA-TXNIP). Therefore, triggering such responses in cancer cells using small molecule compounds may have therapeutic potentials. It is of interest to investigate the mechanisms by which the lactic acidosis response is sensed and triggered in cancer cells. Extracellular lactate enters the cells through the lactate transporter proteins of monocarboxylate transporter (MCT), which may be also important for the lactic acidosis response. Lactate has also been recognized for its role as an energy source [63] and a signaling molecule to affect tumor cell phenotypes and target of cancer therapeutics [97]. Previous studies have shown that lowering the extracellular pH from 7.4 to ∼6.7 will lead to a slight lowering of intracellular pH (pHi) from 7.4 to 6.9–7.0 [98], [99]. Since many surface or cytosolic molecules exhibit high sensitivity to pH, this drop in pHe and the corresponding slight decrease in pHi may induce conformational changes to trigger signaling events [100], [101]. For example, it has been postulated that inhibition of the acidosis-sensing glutamine pumps leads to amino acid depletion [73], [74] and increases in the proteosome activity [102] in muscle cells, also contributing to muscle loss during metabolic acidosis. Low pHi has also been shown to directly enhance both DNA-binding and the TBP-interacting capacity of the transcriptional factor Sp1 [103]. Similarly, it is possible that this slight drop in pHi may destabilize the hydrogen bonds in several histone residues in the basic regions of MondoA to causes its transcriptional activation of target genes [57]. Homeostasis of pHi is mainly regulated by the sodium/hydrogen exchanger (NHE) family of proteins [104], [105] and such regulation is dysregulated in cancer cells [106]. TXNIP is known to be transcriptionally regulated by a variety of stresses and stimuli [84], [87], [107] and it is possible that some of these stimuli may act through modulating pHi to affect MondoA. TXNIP has been proposed as an inhibitor of cell growth [107] and the inhibition of the growth-inhibiting TXNIP expression under alkaline pHi may help to explain its permissiveness for cellular proliferation in response to growth factors [108], [109]. In addition, evidence is accumulating for the role of membrane acid-sensing receptors in either GPR4 family of G-protein coupled receptors (GPCR) and Acid-Sensing Ion Channels (ASICs) in many cell types. These acid-sensing receptors may also be critical in the observed response in both the AMPK-mTOR and MondoA-TXNIP under lactic acidosis. Importantly, these acid-sensing receptors can be modulated by small compounds pharmacologically [110], [111] and thus have potential as cancer therapeutics [112], [113]. For instance, several glycolipids are known natural ligands of these acid-sensing GPCRs and can activate or inhibit receptor responses to acidosis. Small compounds blocking the ASICs have been used to improve the symptoms and severity of stroke after vascular blockage [114]. Similarly, the use of compounds modulating the lactic acidosis response may mimic the tumor suppressor activities of lactic acidosis in novel cancer therapeutics. The definition of key genes and pathways will allow the use of genetic and chemical means to identify how to modulate cellular lactic acidosis for therapeutic purposes and thus potentially improve outcomes for cancer patients.

## Materials and Methods

### Cell culture and conditions modeling different tumor microenvironmental stresses

MCF7 breast cancer cell lines were cultured in DMEM (GIBCO11995) with 4.5 g/L glucose, supplemented with 10% fetal bovine serum, 1× non-essential amino acid and 1× antibiotics (penicillin, 10000UI/ml; streptomycin, 10000UI/ml). MEF cells were cultured in DMEM media supplemented with 15% FBS. Lactic acidosis conditions were created with the addition of 25mM or 10mM lactic acid (Sigma) to respective media and adjusted to the desired pHs with HCL or NaOH. Glucose deprivation was created by using 0 glucose/L media (GIBCO11966). Hypoxia was created by lowering the oxygen level to 1% oxygen. To stabilize the pH of DMEM media better, 25mM HEPES was also added into the media.

### RNA isolation and microarray analysis

RNAs from MCF7 cells exposed to control or lactic acidosis culture conditions for 1, 4, 12, 24 hr were collected at respective time points and extracted with miRVana kits (Ambion), followed by hybridization to Affymetrix Hu133 plus2 gene chips with standard protocols. RNAs from MCF7 cells exposed to lactic acidosis, glucose deprivation and hypoxia were extracted after 4 hour exposure with miRVana kits (Ambion) and hybridized to Affymetrix Hu133 plus2 gene chips in a similar fashion. CEL files were normalized by RMA using Expression console (Affymetrix), filtered by indicated criteria, clustered with cluster 3.0, and displayed with treeview. All the microarray results have been submitted into GEO with accession number GSE19123. The RNA from the treated MEF cells were interrogated by hybridization to Affymetrix mouse 430A2 GeneChip with standard protocols and processed in a similar fashion.

### Statistical analyses

Gene expression signatures associated with 1,4,12, and 24 hour lactic acidosis treatments were derived from RMA expression values using standard Bayesian sparse multivariate regression techniques, full details of which appear in previous publications [18], [115]–[117] For each treatment group (i.e. 1-,4-,12-, and 24-hr lactic acidosis treatment), this estimated the probability of differential expression for each Affymetrix probe as well as the fold change of the differentially expressed transcripts, as compared to the control. These values define the *in-vitro* signature of the treatment. Projection of these signatures into the primary tumor data sets is derived from the weighted inner product of the vector of tumor gene expression values and the vector of regression coefficients associated with the signature, as described in [117]: the score associated with signature k in sample i is defined as 
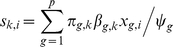
, where 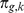 is the estimated probability of differential expression of probe g in treatment k, 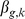 is the estimated fold change of the transcript, 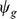 is the estimated residual variance of probe g, and 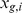 is the RMA normalized expression value of probe g in sample i. The signature score thus provides a relative measure of the extent to which the pattern of expression described by the signature is present or reversed in a given vector of expression values.

P-values associated with the significance of Kaplan Meier curves were derived from survival analyses conducted using a Cox proportional hazards model in MATLAB. Kaplan Meier curves show the optimal stratification of high-risk and low-risk groups as determined by choosing the partition threshold that maximizes the area between curves. Measures of correlation and the associated P-values for the signature score scatter plots were calculated using the Pearson coefficient.

Full details of statistical analysis, including data and computer code to replicate the analyses, are available as Dataset S1.

### Realtime RT–PCR

RNAs were reverse-transcribed to cDNAs with SuperScript II reverse transcription kit following the manufacturer's protocol (Invitrogen). cDNAs were then used as the substrate for gene expression level measured by qPCR with Power SYBRGreen PCR Mix (Applied Biosystem) and primers specific for TXNIP (Forward: CTGGCGTAAGCTTTTCAAGG, Reverse: AGTGCACAAAGGGGAAACAC), ARRDC4 (Forward: CCCCCTCCCACATGGTCACA, Reverse: TCCCTGGCTCCCTTCCATGTGT), Actin-beta (Forward: CTCTTCCAGCCTTCCTTCCT, Reverse: AGCACTGTGTTGGCGTACAG) following the manufacturer's protocol.

### Glucose uptake assays

MCF7 cells were plated in 6-well/12-well plates at the density of 800,000/200,000cells per well. Once cells were more than 75% confluent, they were washed with 1× PBS twice, followed by application of serum starvation media (0.1%FBS) for three hours. Cells were then treated with respective conditions for the desired time. For MEF cells, they were plated in 12-well plates at the density of 100,000 cells per well. Once they reached more than 70% confluence, applying respective indicated conditions for four hours. For glucose uptake measurement, cells would then be washed with 37°C KRH buffer twice, followed by adding in 500ul/200ul KRH buffer containing 0.5uCi/0.2uCi 2-deoxy-D-glucose (GE Healthcare) for one hour in 37°C incubator. 20uM cytochalasin B was added for negative controls. After incubation, cells would be washed three times with 1ml/400ul of ice-cold KRH buffer containing 20mM glucose and 0.5mM phloretin to quench the glucose uptake. Finally, cells were lysed with 1ml/400ul RIPA buffer and the lysates were subjected to liquid scintillation counting. Protein concentrations were measured with Bradford assay. To measure the glucose uptake of genetically-manipulated cells, glucose uptake was measured 24 or 48 hours after the transfections.

### RNA interference

MCF7 cells were plated in 12-well plates for the density of 200,000 cells per well. Once the cells reached 60% confluence, 100nM siRNAs were transfected by using lipofectamine. To verify the successful knocking down of the intended transcripts, RNAs were collected with miRVana kit 24 hours after the transfection, whereas 48 hours after the transfection, proteins were collected by lysing the cells with RIPA buffer.

### Western blot analysis

Proteins were collected with RIPA buffer and their concentrations were measured with Bradford assay. Equal amounts of proteins were loaded for the protein analyses. Primary antibodies of AMPK, S6K (cell signaling), TXNIP (MBL) and anti-MondoA antibodies were applied following the manufacturers' protocols or as described previously [57].

### Cell proliferation assay

Wild type mouse embryo fibroblast (MEF) and TXNIP null MEF cells were plated at the density of 25,000 cells per ml. The multi-channel pipette was used to plate 100ul of cell suspension evenly into the 96-well cell culture plate. Respective conditions of 10mM or 25mM lactic acidosis were applied the next day and cell number was estimation by using a standard MTS (3-(4,5-dimethylthiazol-2-yl)-5-(3-carboxymethoxyphenyl)-2-(4-sulphophenyl)-2H-tetrazolium)colorimetric assay (Promega) 48 hours after respective treatments.

### Chromatin Immunoprecipitation

ChIP studies were performed as described previously [57] using an off-target region located on chromosome 10 to calculate fold enrichment. Primer sequences for the promoters of TXNIP, ARRDC4, and the off target control of the CHIP analysis are available upon request to D.E.A.

### Glucose and lactate measurement in media

MCF cells were plated in six-well plates at a density of 500,000 cells per well. The next day, non-targeting siRNAs and siRNAs against TXNIP were transfected as described previously. After 24 hours, fresh media for the respective conditions, including control and 25mM lactic acidosis were applied to cells for 48 hrs when media were collected for glucose (ACCU-CHECK Aviva, Roche) and lactate (ARKRAY) measurements with respective meters. The results were normalized by cell numbers to obtain the glucose consumption and the lactate production amount per million cells.

## Supporting Information

Dataset S1 Statistical code and other supporting material contains data, code, and information on the statistical analysis of in vitro expression data to generate signatures, as well as of in vivo projection of signatures into the sets of human breast cancer data. Analysis uses the BFRM software previously described and used in multiple related studies [115] and freely available, with tutorial examples. Supplementary material contains a readme describing the setup and running of the analyses.
(0.03 MB ZIP)

Figure S1 The prognostic values of gene signatures reflecting lactic acidosis response at different time points among the patients in different cancer expression datasets. The graphs show Kaplan-Meier curves for two patient subsets stratified by the level of lactic acidosis response. The p-values are for regression coefficients of the signature in the survival model analysis.
(0.27 MB PDF)

Figure S2 Scatter plots showing the relationship between the levels of lactic acidosis response as defined by HMEC (24hrs) (Y-axis) and MCF-7 at different time points of lactic acidosis exposure (X-axis). Each point in the scatter plots represents a single tumor from the indicated breast cancer data sets. The overall correlation (R) and statistical significance/p-value (p) between the predicted lactic acidosis pathway activities using these two breast cancer cells across all samples is shown for the indicated data set.
(0.55 MB PDF)

Figure S3 The prognostic significance of the 109 genes (assayed in the Miller datasets out of the 115 genes) which are affected in opposite directions by lactic acidosis and glucose deprivation (LA/GD). (B) The prognostic significance of three TXNIP probsets in the Miller datasets. (C) The prognostic significance of the signature of the 106 genes after the removal of the three TXNIP probesets in the Miller dataset.
(5.02 MB EPS)

Figure S4 Heatmap showing the upregulation of TXNIP and ARRDC4 in MCF-7 and HMECs at different time points of exposure to lactic acidosis from the microarray analysis.
(0.21 MB PDF)

Figure S5 Realtime RT-PCR results of ARRDC4 expression normalized by b-actin under control, lactic acidosis, glucose deprivation, and hypoxia.
(0.24 MB PDF)

Figure S6 The induction of TXNIP in WiDr and SiHa cells under lactic acidosis.
(0.28 MB PDF)

Figure S7 The measured glucose consumption (A) and lactate production (B) of the MCF-7 which has been transfected with indicated siRNAs either non-targeting (-) and TXNIP (T) under control and lactic acidosis conditions.
(0.31 MB PDF)

Figure S8 The effect of TXNIP disruption on the gene expression under control and 10mM lactic acidosis conditions. 798 probes sets showing with at least 1.7-fold changes in at least two samples were selected and arranged by hierarchical clustering according to similarities in expression patterns with the names of selected genes shown.
(0.29 MB PDF)

Figure S9 The pair-wise t-test and p value for the comparison of the 1048 repressed genes (A) and 277 induced genes (B) between the TXNIP deficient and wild-type littermate MEF cells based on the lactic acidosis gene expression derived by zero-transformation.
(0.62 MB PDF)

Figure S10 The amount (%) of lactic acidosis-induced repression in glucose uptake of the MCF-7 which has been transfected with indicated siRNAs either non-targeting (-), MondoA (M1, M2).
(0.22 MB PDF)

Table S1 The pathway composition analyzed by GSEA in the MCF-7 exposed to lactic acidosis versus normal conditions for samples in all time points.
(0.02 MB XLS)

Table S2 The probesets and average folds of change in affected by indicated conditions are shown for the 115 probe sets which were affected in opposite direction by lactic acidosis and glucose deprivation with the top 1% probability.
(0.01 MB XLSX)
