# Supplementary figures and images for: Lactic Acidosis Triggers Starvation Response with Paradoxical Induction of TXNIP through MondoA
Source: PLoS Genet. 2010 Sep 2;6(9):e1001093. doi: 10.1371/journal.pgen.1001093 (PMC2937306; doi:10.1371/journal.pgen.1001093)

A.Pawitan

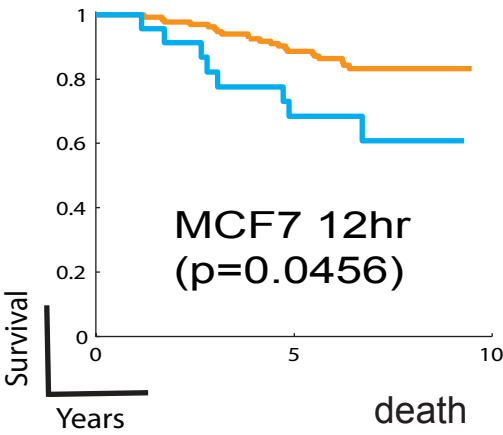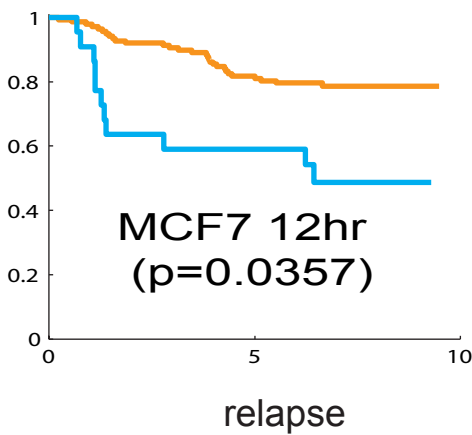

B.Sotiriou

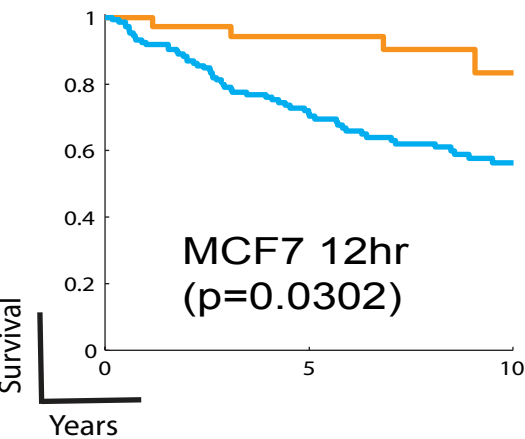

High LacAcid  
Low LacAcid

C.Wang

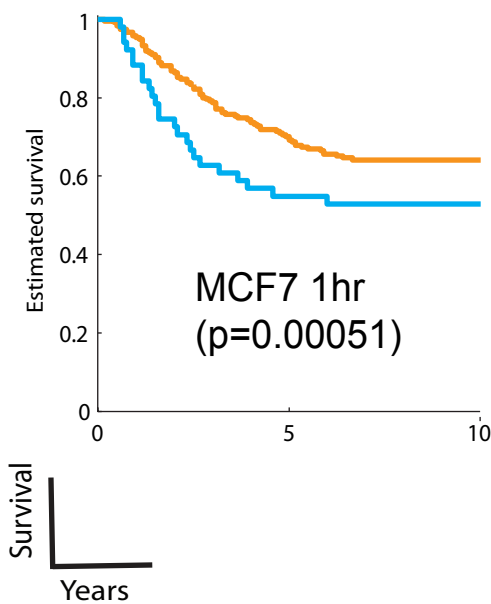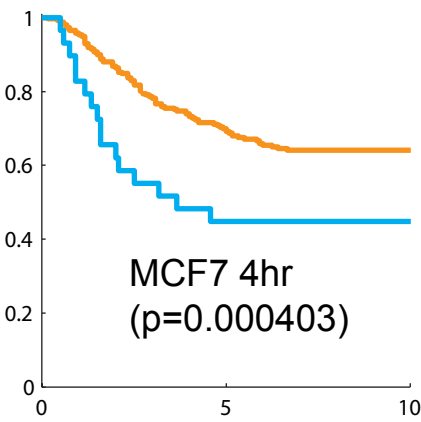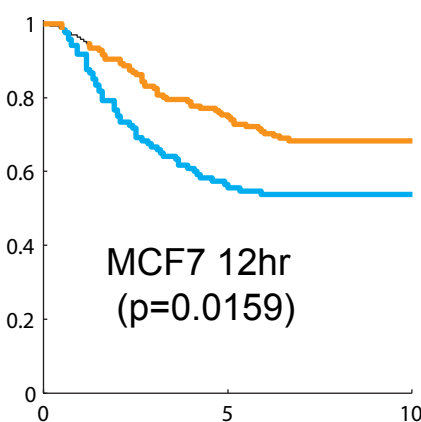

Supplement: Figure S1 — The prognostic values of gene signatures reflecting lactic acidosis response at different time points among the patients in different cancer expression datasets. The graphs show Kaplan-Meier curves for two patient subsets stratified by the level of lactic acidosis response. The p-values are for regression coefficients of the signature in the survival model analysis. (0.27 MB PDF) [file pgen.1001093.s002.pdf]

### A. Sotiriou

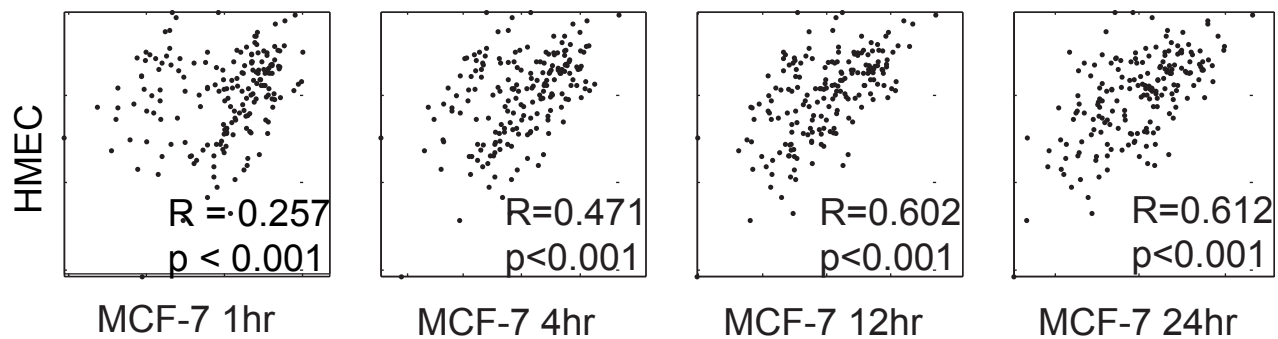

### B. Pawitan

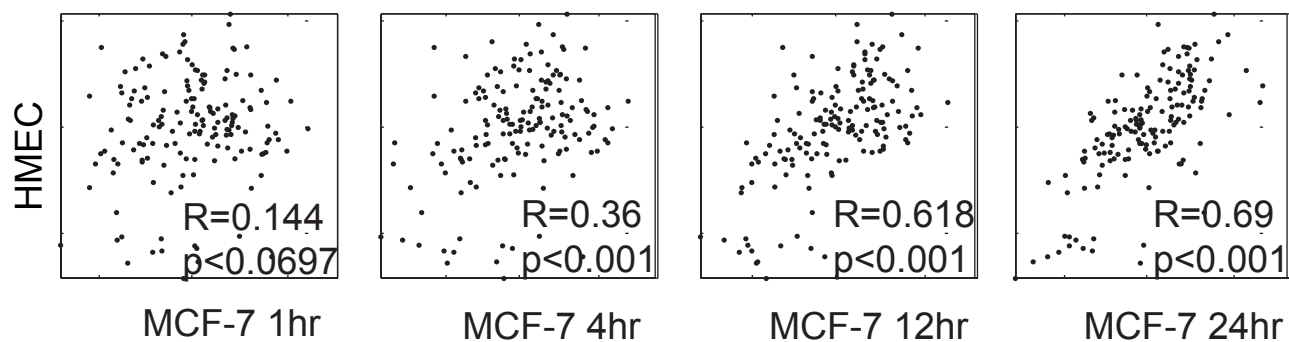

### C. Wang

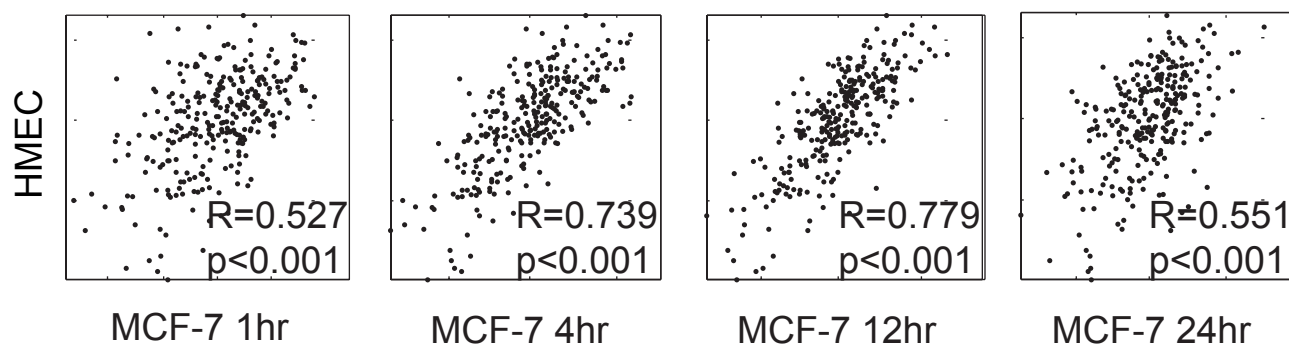

Supplement: Figure S2 — Scatter plots showing the relationship between the levels of lactic acidosis response as defined by HMEC (24hrs) (Y-axis) and MCF-7 at different time points of lactic acidosis exposure (X-axis). Each point in the scatter plots represents a single tumor from the indicated breast cancer data sets. The overall correlation (R) and statistical significance/p-value (p) between the predicted lactic acidosis pathway activities using these two breast cancer cells across all samples is shown for the indicated data set. (0.55 MB PDF) [file pgen.1001093.s003.pdf]

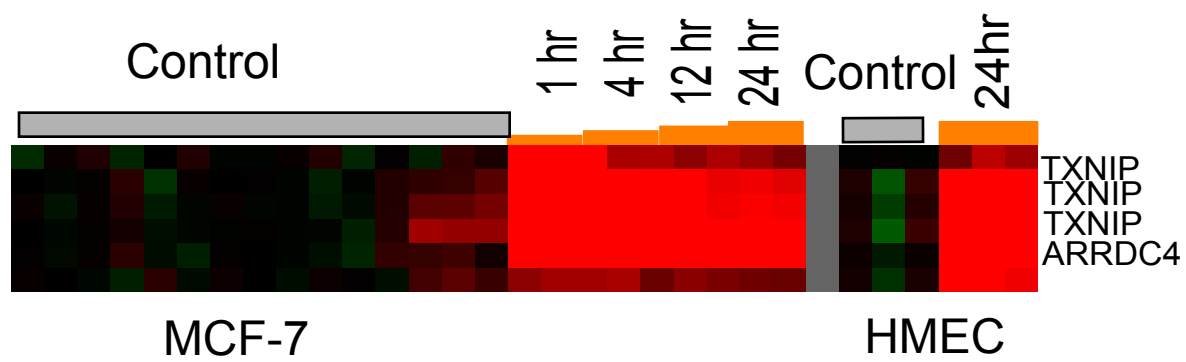

Supplement: Figure S4 — Heatmap showing the upregulation of TXNIP and ARRDC4 in MCF-7 and HMECs at different time points of exposure to lactic acidosis from the microarray analysis. (0.21 MB PDF) [file pgen.1001093.s005.pdf]

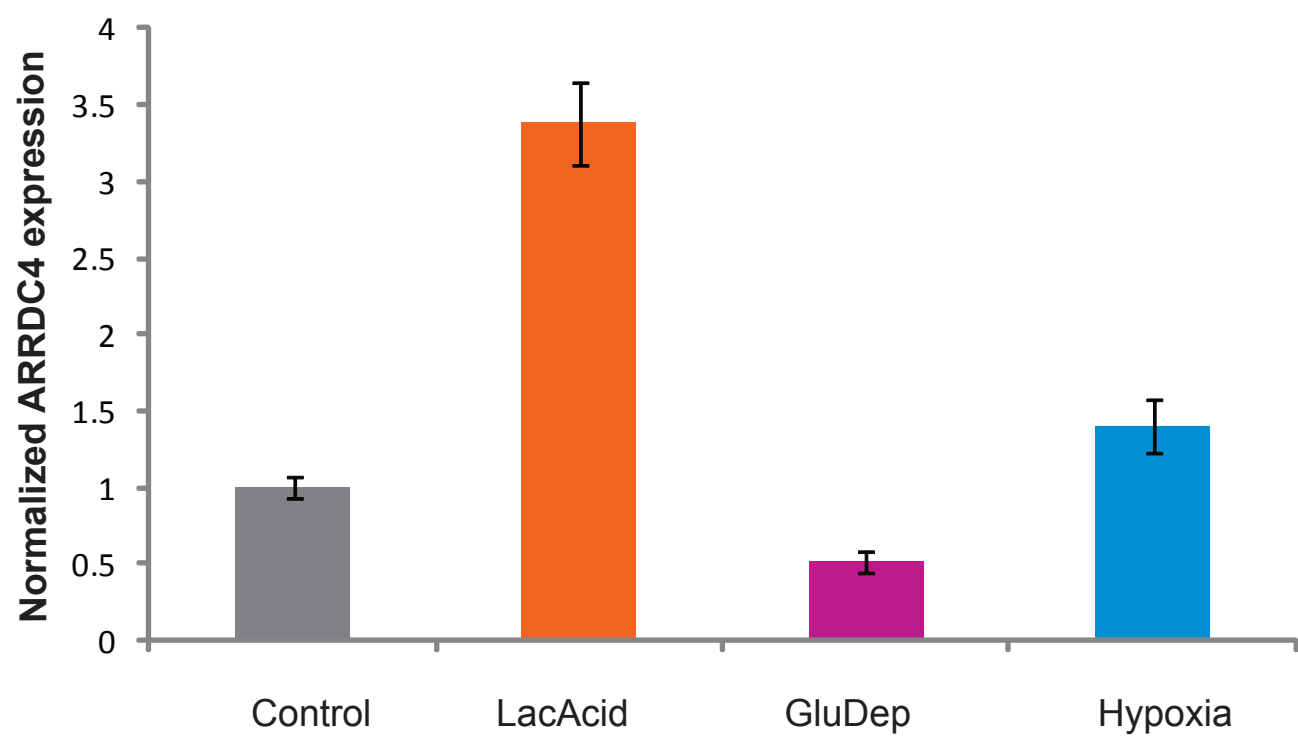

Supplement: Figure S5 — Realtime RT-PCR results of ARRDC4 expression normalized by b-actin under control, lactic acidosis, glucose deprivation, and hypoxia. (0.24 MB PDF) [file pgen.1001093.s006.pdf]

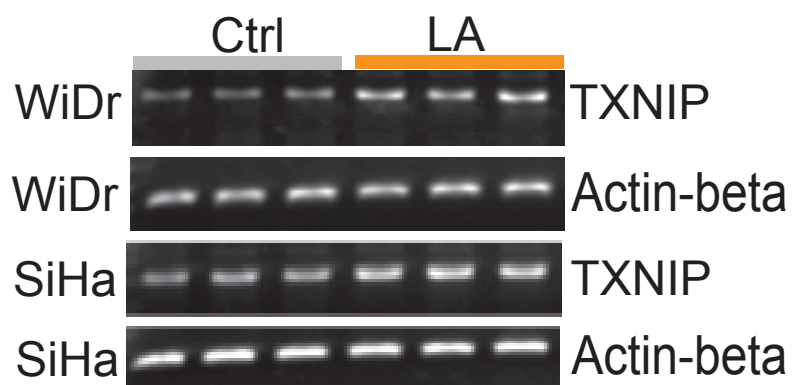

Supplement: Figure S6 — The induction of TXNIP in WiDr and SiHa cells under lactic acidosis. (0.28 MB PDF) [file pgen.1001093.s007.pdf]

# Glucose consumption

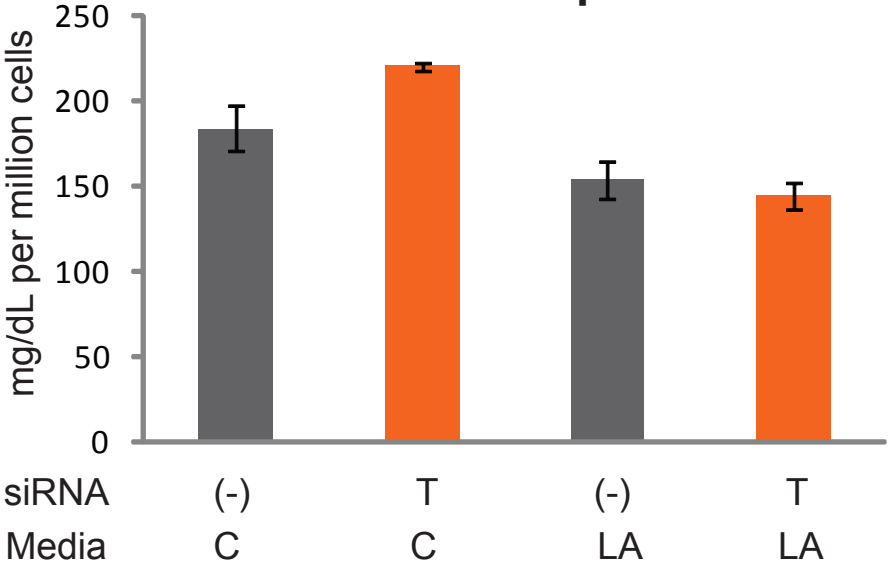

# Lactate production

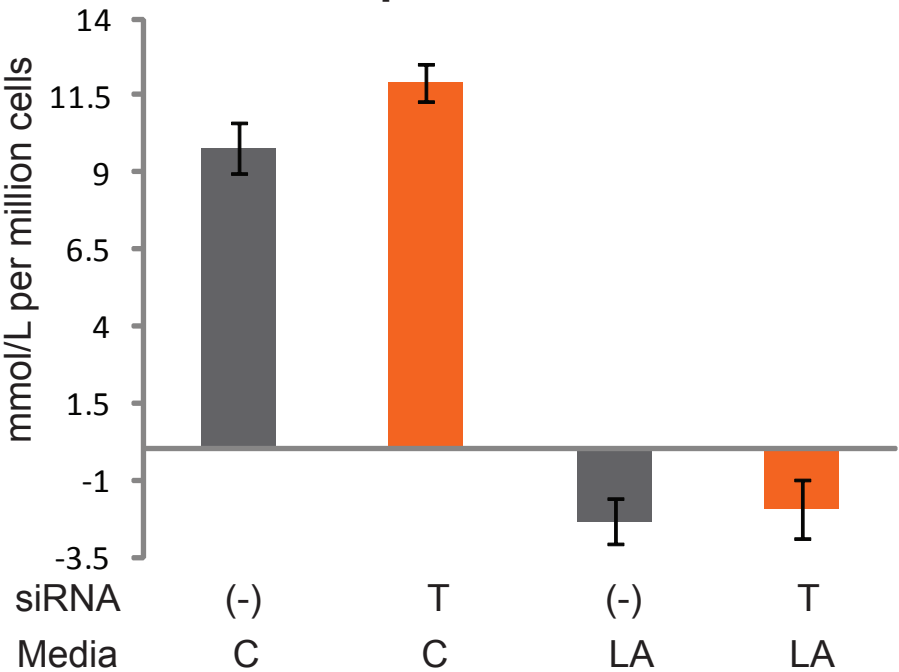

Supplement: Figure S7 — The measured glucose consumption (A) and lactate production (B) of the MCF-7 which has been transfected with indicated siRNAs either non-targeting (-) and TXNIP (T) under control and lactic acidosis conditions. (0.31 MB PDF) [file pgen.1001093.s008.pdf]

Control LA

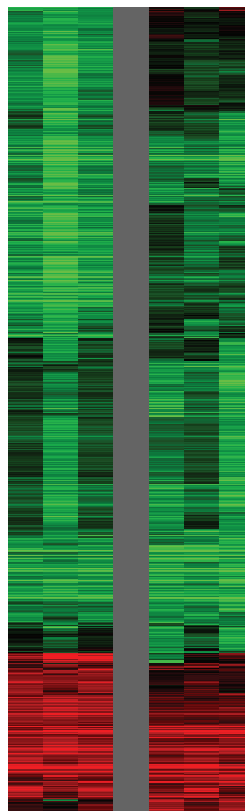

Collagen I  
Collagen 2

TXNIP  
HoxC6  
COX2

C1qc  
C1qb  
AP2  
Notch3

Supplement: Figure S8 — The effect of TXNIP disruption on the gene expression under control and 10mM lactic acidosis conditions. 798 probes sets showing with at least 1.7-fold changes in at least two samples were selected and arranged by hierarchical clustering according to similarities in expression patterns with the names of selected genes shown. (0.29 MB PDF) [file pgen.1001093.s009.pdf]

A.

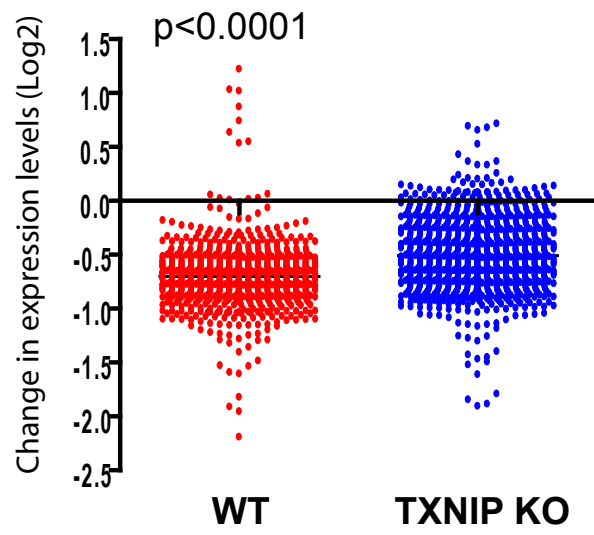

B.

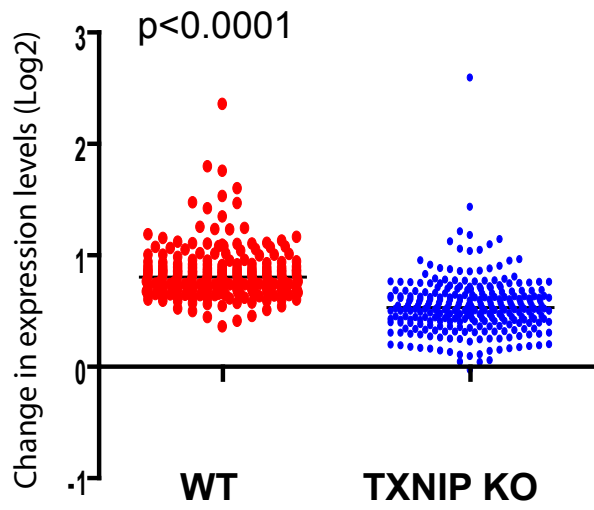

Supplement: Figure S9 — The pair-wise t-test and p value for the comparison of the 1048 repressed genes (A) and 277 induced genes (B) between the TXNIP deficient and wild-type littermate MEF cells based on the lactic acidosis gene expression derived by zero-transformation. (0.62 MB PDF) [file pgen.1001093.s010.pdf]

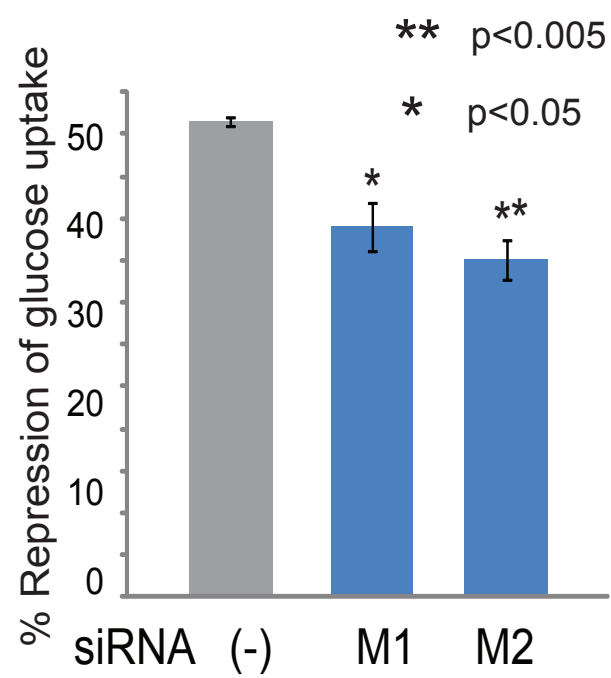

Supplement: Figure S10 — The amount (%) of lactic acidosis-induced repression in glucose uptake of the MCF-7 which has been transfected with indicated siRNAs either non-targeting (-), MondoA (M1, M2). (0.22 MB PDF) [file pgen.1001093.s011.pdf]
